# Overexpression of lily MicroRNA156-resistant *SPL13A* stimulates stem elongation and flowering in *Lilium formosanum* under non-inductive (non-chilling) conditions

**DOI:** 10.3389/fpls.2024.1456183

**Published:** 2024-10-18

**Authors:** Masumi Yamagishi, Toshikazu Nomizu, Takashi Nakatsuka

**Affiliations:** ^1^ Research Faculty of Agriculture, Hokkaido University, Sapporo, Japan; ^2^ Biotechnology Division, Niigata Agricultural Research Institute, Nagaoka, Niigata, Japan; ^3^ Faculty of Agriculture, Shizuoka University, Shizuoka, Japan; ^4^ College of Agriculture, Academic Institute, Shizuoka University, Shizuoka, Japan

**Keywords:** age pathway underlying floral transition, leaf angle, microRNA156/SPL module, microRNA172, SQUAMOSA promoter-binding protein-like, transitions from a juvenile to an adult vegetative phase

## Abstract

Flowering plants undergo juvenile vegetative, adult vegetative, and reproductive phases. Lily plants (*Lilium* spp.) develop scaly leaves during their juvenile vegetative phase. Stem elongation occurs in the adult vegetative phase and is followed by floral transition. As the duration of the juvenile vegetative phase is long in lilies, the microRNA156 (miR156) and SQUAMOSA PROMOTER-BINDING PROTEIN-LIKE (SPL) modules are expected to play a major role in vegetative phase change and flower induction. In the present study, we aimed to explore the functions of lily SLP13A. We evaluated phenotypic changes and gene expression in *L. formosanum* plants overexpressing miR156-resistant *SPL13A* (*rSPL13A*) and examined the accumulation levels of gene transcripts and mature miRNAs in non-transformed *L. longiflorum* plants. Lily plants overexpressing *rSPL13A* exhibited stem elongation under non-inductive conditions, and FLOWERING LOCUS T (*FT*) genes were poorly involved in this stem elongation. Flowering was induced in the transformed plants with elongated stems, and the accumulation of *MADS5* (APETALA1) transcripts and mature miR172 was elevated in these plants. In non-transformed lilies, *SPL13A* transcripts were highly accumulated in the shoot apices of both juvenile and adult plants. As mature miR156 was poorly accumulated in the shoot apices of the adult plants, SPL13A was active enough to stimulate stem elongation and flower induction. In contrast, mature miR156 was reliably detected in shoot apices of the juvenile plants. Because our transient assay using tobacco plants expressing a SPL13A-GFP fusion protein indicated that miR156 repressed *SPL13A* expression mainly at the translational level, SPL13A activity should be insufficient to stimulate stem elongation in the juvenile plants. In addition, the accumulation of *MADS5* transcripts and mature miR172 in the shoot apices increased with plant growth and peaked before the transition to the reproductive phase. Therefore, we conclude that SPL13A regulates stem elongation in the adult vegetative phase, which differs from the mechanisms evaluated in Arabidopsis and rice, wherein stem elongation proceeds in a reproductive phase and *FT* genes are heavily involved in it, and that *SPL13A* induces flowering by the activation of genes related to the age pathway underlying floral transition, as APETALA1 and *primary-MIR172* are mainly involved in this pathway.

## 1 Introduction

Flowering plants undergo juvenile vegetative, adult vegetative, and reproductive phases. Most plants do not develop flowers during the juvenile vegetative phase, even if they are grown under conditions that support floral transition and floral meristem identity (flower induction). After the shoot transitions from the juvenile to adult vegetative phase (vegetative phase change), plants acquire competence for flowering and progress to the reproductive phase by integrating a wide range of environmental and developmental cues (Wang, 2014; Hyun et al., 2017). The microRNA156 (miR156) and SQUAMOSA PROMOTER-BINDING PROTEIN-LIKE (SPL) module mainly regulate vegetative phase changes (Poethig and Fouracre, 2024). The levels of miR156 accumulation are high during the juvenile vegetative phase and decline as the shoots mature. As *SPL* genes are targeted by miR156, their activities increase with decreasing miR156 levels. Along with vegetative phase changes, the size, shape, and surface structures (including wax and trichomes) of leaf blades and petiole length are altered, and the miR156/SPL module is strongly involved in these alterations (Chuck et al., 2007a; Wang, 2014; Hu et al., 2023). Arabidopsis has 16 *SPL* genes that are classified into eight clades (clade I–VIII, Preston and Hileman, 2013). Of these, ten *SPLs*, namely, *SPL2/10/11* (clade V), *SPL3/4/5* (clade VI), *SPL6* (clade IV), *SPL9/15* (clade VIII), and *SPL13* (clade VII), are negatively regulated by miR156. SPL2/10/11, SPL9/15, and SPL13 contribute to vegetative phase change, floral transition, and floral meristem identity, and SPL9/15 and SPL13 play more dominant roles than SPL2/10/11. SPL3/4/5 are mainly responsible for the floral meristem identity (Xu et al., 2016). In addition to their roles in phase transition and flower induction, SPLs often play additional roles in other species. For example, rice SPLs, including OsSPL14 (IPA1, clade VIII) and OsSPL16 (GW8, clade VII), regulate panicle branching and grain shape (Jiao et al., 2010; Miura et al., 2010; Wang et al., 2012; Wang and Zhang, 2017). In tomatoes, SlSPL13 regulates inflorescence morphogenesis, lateral branch development, and fruit yield (Cui et al., 2020). As these additional roles vary depending on the species, the functions of *SPLs* must be characterized for each species to determine their unique roles.

The six regulatory pathways underlying floral transition, namely, the age, vernalization, photoperiod, ambient temperature, autonomous, and gibberellin (GA) pathways, have been elucidated mainly in Arabidopsis (Liu et al., 2009; Srikanth and Schmid, 2011). The main gene expression cascade in the age pathway is as follows: After a decline in miR156 accumulation, SPLs activate the expression of floral meristem identity genes, including APETALA1 (*AP1*)/SQUAMOSA (*SQUA*) (Wang et al., 2009; Hyun et al., 2016) and floral transition genes, including *primary MIR172* (Wu et al., 2009; Wang, 2014). miR172 enhances floral transition because APETALA2 (*AP2*) and *AP2-like* genes, which are flower induction repressors, are targeted by miR172 (Mathieu et al., 2009; Ó’Maoiléidigh et al., 2021). In the vernalization pathway, prolonged exposure to low temperatures activates the expression of FLOWERING LOCUS T (*FT*) and SUPPRESSOR OF OVEREXPRESSION OF CO 1 (*SOC1*), which are the major genes involved in floral transition (Madrid et al., 2021).

Cultivated lilies are among the most economically important floricultural plants worldwide, and most cultivars have been bred using interspecific hybridization (Marasek-Ciolakowska et al., 2018). For example, longiflorum hybrids (*L*. × *formolongi*) are derived from the hybridization of *L. formosanum* × *L. longiflorum*, both of which belong to the Leucolirion b section. Asiatic hybrids are developed by crosses among species of the Sinomartagon section. Clarifying the endogenous and environmental factors that affect lily growth and flower induction is valuable for improving cultivation conditions and enhancing breeding. The chilling (vernalization) of bulbs is necessary to induce stem elongation and flowering in most lilies (Mazor et al., 2021). One notable exception is the small bulbs of *L. longiflorum*, which flower effectively under long day conditions without chilling. Large bulbs of *L. longiflorum*, like most other lilies, require vernalization for stem elongation and flower induction (Lazare and Zaccai, 2016). Gene functions involved in flower induction have been characterized in lilies. Lily MADS BOX GENE 5 (*MADS5*), *MADS6*, and *MADS7* have similar functions to *AP1* and FRUITFULL in Arabidopsis (Chen et al., 2008). *FT* is one of the most studied genes controlling flowering time in lilies. Among the *FT-like* genes in Asiatic hybrid lilies, *FT1* and *FT8* have high sequence homology to *Hd3a* in rice and *FT* in Arabidopsis, respectively (Kurokawa et al., 2020). *Lilium longiflorum FT* (*LlFT*) might be an ortholog of *FT8* in Asiatic hybrid lilies, and the overexpression of *LlFT* in *L. longiflorum* results in early flowering under non-inductive conditions (Leeggangers et al., 2018). Ectopic overexpression of *FT1* complements the late-flowering phenotype of the Arabidopsis *ft-10* mutant, whereas overexpression of *FT8* does so to a lesser degree. *FT1* is mainly expressed in the bulb scales after vernalization, whereas *FT8* is strongly expressed in the bulb scales during cold exposure (Kurokawa et al., 2020). Thus, *FT1* and *FT8* (*LlFT*) should be major regulators of the vernalization pathway in lilies. In comparison with that of *FT8*, the temporal profile of *FT1* expression is well correlated with early- and late-flowering behaviors in an Asiatic hybrid lily cultivar and *in L. leichtlinii*, which is a species used for Asiatic hybrid lily establishment (Kurokawa et al., 2020). *FT1* and *FT8* are expressed mainly in the bulb scales (underground organs), which does not contradict the fact that day length usually has a small effect on flower induction. However, evaluation of the regulatory pathways underlying flower induction other than the vernalization pathway is limited in lilies. The functions of other floral integrator genes, including *SPL* and *SOC1*, and floral meristem identity genes, including LEAFY, have not yet been elucidated in lilies, although RNA-seq analyses have revealed the transcriptome profiling of such gene sequences (Lugassi-Ben Hamo et al., 2015; Villacorta-Martin et al., 2015; Li et al., 2016, 2017).

Upon germination, the shoot apices of the lilies develop scaly leaves, the basal portions of which swell and produce bulb scales. Over several months (for Longiflorum hybrids) or years (for most lily species and cultivars), lily shoots develop only scaly leaves without stem elongation resulting in bulb enlargement. Stem elongation begins after the scaly leaf differentiation ceases. Cold exposure of the bulb is necessary to initiate stem elongation (Dole and Wilkins, 1994; Lugassi-Ben Hamo et al., 2015). Although the growth stages corresponding to a juvenile vegetative phase or an adult vegetative phase have not been well defined in lilies, it is thought that lily shoots that differentiate into scaly leaves are in a juvenile vegetative phase because flower induction never occurs when scaly leaves are developing, and that lily plants elongating stems are in an adult phase. Conversion of the shoot apical meristem to an inflorescence meristem (floral transition) occurs at the apices of elongating stems. Stem elongation is followed by floral transition in most lily species and hybrids (Roh and Wilkins, 1977; Leeggangers et al., 2018). Because several years are usually necessary from sowing to the initiation of stem elongation, lilies are thought to have a long juvenile vegetative phase. Thus, the miR156/SPL module is expected to play a significant role in the lily life cycle. Clarifying the internal and external factors affecting the length of the juvenile vegetative phase enables the breeding of novel lily cultivars exhibiting shorter duration of flowering from sowing; these cultivars include those that can be propagated by seeds, such as longiflorum hybrids, which can flower within a year after sowing (Anderson, 2012).

*The SPL* genes of clade VII play significant roles in phase changes and development in Arabidopsis, rice, and alfalfa (Wang et al., 2012; Xu et al., 2016; Gao et al., 2018; Yuan et al., 2019): In the current study, the functions of lily SPL13A belonging to clade VII were evaluated. SPLs often have functions unique to each species, and a reverse genetics approach is necessary to detect such unique functions: Phenotype and gene expression were evaluated in transgenic *L. formosanum* plants overexpressing the miR156-resistant *SPL13A* (*rSPL13A*) gene. The accumulation of miR156 and miR172, and the transcription of *SPL13A* and other genes related to flower induction were evaluated during the growth of *L. longiflorum* plants, a species closely related to *L. formosanum*. Effects of miR156 on *SPL13A* expression were also evaluated using transient assays to determine whether miR156 targets SPL13A in lilies. We found that lily *SPL13A* was involved in stem elongation during the adult vegetative phase and flower induction, suggesting that SPL plays a unique role in stem elongation in *Lilium*.

## 2 Materials and methods

### 2.1 Plant materials

For the lily transformation, *L. formosanum* var. *pricei* was used because of its high transformation efficiency (unpublished result). To evaluate the gene expression and miRNA accumulation in non-transformed (wild type) lily plants, *L. longiflorum* cv. ‘White Heaven’ was used. Large bulbs (> 7 cm in diameter) of ‘White Heaven,’ which were frozen at -1.5 – -2.0°C and then thawed under a couple of temperature conditions, were purchased from Takii Seed Co. (Kyoto, Japan). After receiving the bulbs in late June, they were immediately planted in pots filled with soil (a mixture of clay loam soil and leaf mold [2:1]) with fertilizer, and grown in a greenhouse (unheated and natural photoperiod) at the experimental farm of Hokkaido University, Sapporo, Japan. Roots, basal plates, inner and outer bulb scales, shoot apices, stems, leaves, and daughter bulbs were harvested at 0, 1, 2, 3, 4, 5, 6, and 7 weeks after planting and used for gene expression analysis. Scale propagation was initiated in late June, to obtain small plants. Roots, basal plates, bulb scales, shoot apices, and leaves were harvested in early November (17 weeks later) from newly developed bulblets (< 1 cm in diameter) and developing scaly leaves (without stem elongation).

To analyze the effects of plant age, bulblets (< 1 cm in diameter) detached from the mother bulb scales (scale propagation) and large bulbs (> 7 cm in diameter) were stored at 4°C for 16 weeks, planted in spring, and cultivated for 2 weeks under the conditions described above. To evaluate the effect of cold exposure, large bulbs (> 7 cm in diameter) were stored at 4°C or 25°C for 8 weeks and then cultivated for 4 weeks under the conditions described above. Inner bulb scales and shoot apices were collected from plants for gene expression analyses.

For agroinfiltration, *Nicotiana benthamiana* plants were grown in a growth chamber at 24°C under a 16-h light-8-h dark photoperiod.

### 2.2 35Sp::rSPL13A construction and lily transformation

The coding region of lily *SPL13A* was PCR-amplified using the cDNA of *L. longiflorum* cv. ‘White Heaven’ and sequenced. To induce mutation at an miR156-binding site (miR156-resistant *SPL13A*, *rSPL13A*), an overlap primer extension PCR technique was used, resulting in that nucleotide sequence at the site was modified from 5′-GTGCTCTCTCTCTTCTGTCA-3′ to 5′-GCGCATTGAGCTTGTTAAGT-3′ without changing an amino acid sequence. All primers used in this study are listed in **Supplementary Table S1**. The amplified products were inserted into the pIG121-Hm vector (accession no. AB489142.1) between the cauliflower mosaic virus 35S promoter (35Sp) and the nopaline synthase terminator, using XbaI and SacI restriction sites (hereinafter, referred to as the 35Sp::rSPL13A construct). The constructs were introduced into *Agrobacterium tumefaciens* strain EHA105 using electroporation. The transformation of *L. formosanum* var. *pricei* was performed following Hoshi et al. (2004) and Satou et al. (2022). Briefly, lily calli were induced from filaments collected from young flower buds (< 2.5 cm in length) on an MgSO_4_-free MS medium supplemented with 2 mg·L^-1^ picloram and 30 g·L^-1^ sucrose. After inoculation of the *Agrobacterium* for 2 days, transformed calli were selected and plants were regenerated on the MS medium containing 0.1 mg·L^-1^ picloram, 30 g·L^-1^ sucrose, and 50 mg·L^-1^ hygromycin. Then, transformed plants were transplanted to culture vessels (6 cm × 6 cm × 9.5 cm height) containing 40 mL hormone free MS medium with 60 g·L^-1^ sucrose. All *in vitro* cultures were carried out at 25°C under continuous dark (for callus culture) or a 16 h-light/8 h-dark cycle (for bulblet culture). Regenerated lily plants were transplanted in 9-cm diameter pots filled with a nursery soil (KUMIAI ENGEI-BAIDO, Hokusan, Kitahiroshima, Hokkaido, Japan) containing fertilizer (374 mg·kg^-1^ N, 1,485 mg·kg^-1^ P_2_O_5_, 242 mg·kg^-1^ K_2_O, and 165 mg·kg^-1^ MgO) and grown at 25°C under a 16 h-light/8 h-dark cycle. Chilling was not applied to the transformed plants during transplantation.

### 2.3 Agrobacterium-mediated transient assay (agroinfiltration)

To confirm the miRNA-mediated target gene repression, we constructed SPL13A or rSPL13A fused with a green fluorescence protein (GFP). The sequences of *SPL13A*, *rSPL13A*, and *GFP* were amplified, and the *SPL13A-GFP* and *rSPL13A-GFP* sequences were amplified using an overlap primer extension PCR procedure. The GGGSGG polypeptide linker, 5′-GGTGGCGGAAGCGGCGGA-3′, was included between *SPL13A* and *GFP*, and *rSPL13A* and *GFP*. The fragments of *SPL13A-GFP* and *rSPL13A-GFP* were inserted into the pBE2113-GUS binary vector (Mitsuhara et al., 1996) using restriction enzymes SacI and XbaI. *Primary-MIR156B* (*AtMIR156B*, AT4G30975) and *precursor-MIR172B* (*AtMIR172B*, MW775398, Werner et al., 2021) in Arabidopsis were used to generate mature miRNAs because the primary- or precursor-microRNA sequences of miR156 and miR172 are not known in *Lilium*. These sequences were amplified using the genomic DNA of Arabidopsis as a template, and the amplified products were inserted into the pIG121-Hm vector using XbaI and SacI restriction sites, as described above. Following the transformation of *A. tumefaciens* strain EHA105 with each of the binary vectors using electroporation, *A. tumefaciens* suspension was infiltrated into the leaves of *N. benthamiana* using a syringe (Suzuki et al., 2015). To detect the GFP signal, a power LED (485 nm) equipped with a 490 nm Short-pass filter was used for irradiation and photographs were taken using a 530/40 nm Bandpass filter (BioTools Inc., Takasaki, Japan). Fluorescent leaf regions were excised and used for RNA extraction.

### 2.4 Gene expression and mature-microRNA accumulation analyses

Low molecular weight RNA (LMW RNA) and normal molecular weight RNA (RNA) were separately isolated from the lily organs using a High Pure miRNA Isolation Kit (Roche Diagnostics K. K., Tokyo, Japan). In an agroinfiltration experiment, RNA was extracted from *N. benthamiana* leaves using a NucleoSpin® RNA Plus kit (MACHEREY–NAGEL GmbH & Co. KG, Düren, Germany).

To estimate miRNA accumulation, mature miR156 and miR172 were synthesized into cDNA by reverse transcribing LMW RNA using stem-loop pulsed RT protocol, then quantitative end-point PCR was performed to amplify 60-nt fragments that included the mature miRNA sequences (Varkonyi-Gasic et al., 2007). To confirm the quality of the LMW RNA, U6 small nuclear RNA was amplified by reverse transcribing LMW RNA, followed by quantitative PCR amplification. For the analysis of mRNA transcript accumulation, cDNA was synthesized from RNA using the ReverTraAce® qPCR RT Master Mix with gDNA Remover (Toyobo, Tokyo, Japan).

Quantitative PCR was conducted using the THUNDERBIRD® SYBR® qPCR Mix (Toyobo). Signals were monitored using a CFX Connect Real-Time System (Bio-Rad, Hercules, CA, USA). The amount of lily U6 or lily ACTIN in each sample was used to normalize the amount of each target miRNA or mRNA, respectively, using the formula 2^−ΔCt^, where ΔCt = Ct(target gene) − Ct(reference gene). Neomycin phosphotransferase II (NPTII) was used as the reference gene in the agroinfiltration experiment. Statistical differences were analyzed using Tukey’s honestly significant difference (HSD) test in R v3.3.12.

### 2.5 Phylogenetic analysis

Amino acid sequence alignments and a phylogenetic tree were constructed using the phylogenetic analysis pipeline of ETE3, as implemented in GenomeNet, Kyoto University Bioinformatics Center (https://www.genome.jp/tools/ete/), with default parameters.

## 3 Results

### 3.1 Appearance of transformed lily plants overexpressing a *SPL13A* gene

**Figure 1:**
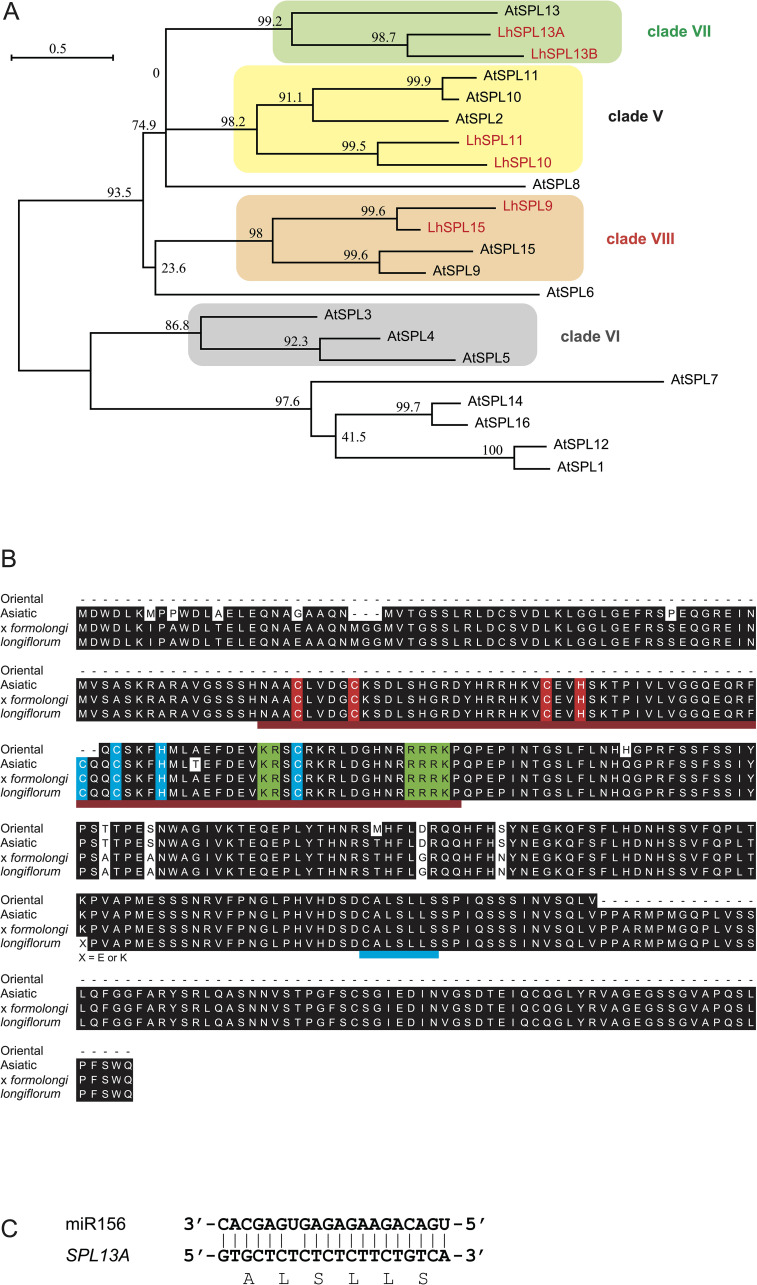
**(A)** A phylogenetic tree of SPL proteins in lilies (red letters) and Arabidopsis. Numbers next to nodes indicate bootstrap value (%). Bar indicates genetic distance 0.5. The accession numbers of Arabidopsis sequences are listed in **Supplementary Table S2**. **(B)** Amino acid sequence alignment of SPL13A in Oriental hybrid lily ‘Dizzy’ (Oriental), Asiatic hybrid lily ‘Lollypop’ (Asiatic), *Lilium* × *formolongi* ‘Raizan 2’ (× *formolongi*), and *L. longiflorum ‘*White Heaven’ (*longiflorum*). The Oriental sequence was partial, and the *L longiflorum* sequences had two heterozygous sequences, in which one amino acid differed. Dark red underlines indicate the SQUAMOSA promoter binding protein domain (Yamasaki et al., 2004) and a blue underline exhibits the miR156 binding region. The two conserved zinc finger structures (Zn-1, red background, and Zn-2, blue background) and NLS (green background) are shown. **(C)** Base pairs between miRNA156 and *SPL13A*.

To investigate the roles of the miR156/SPL module in the vegetative phase change and flower induction of lilies, we screened *SPL* gene sequences using the lily transcriptomes of Asiatic hybrid lily ‘Lollypop’ (Suzuki et al., 2016) and Oriental hybrid lily ‘Dizzy’ (Yamagishi et al., 2018), and isolated several related sequences including those of *SPL13A*. Lily SPL13A showed homology with AtSPL13 (clade VII; **Figure 1A**). The amino acid sequences of four SPL13A in Asiatic hybrid lily ‘Lollypop,’ Oriental hybrid lily ‘Dizzy,’ *L. longiflorum* ‘White Heaven’ (sequenced in this study), and *L*. × *formolongi* ‘Raizan 2’ (found in GenBank database, APY23910.1) indicated that these sequences were highly conserved among *Lilium* species (**Figure 1B**). The miR156 recognition site was located in a coding region (**Figure 1B, C**).

**Figure 2:**
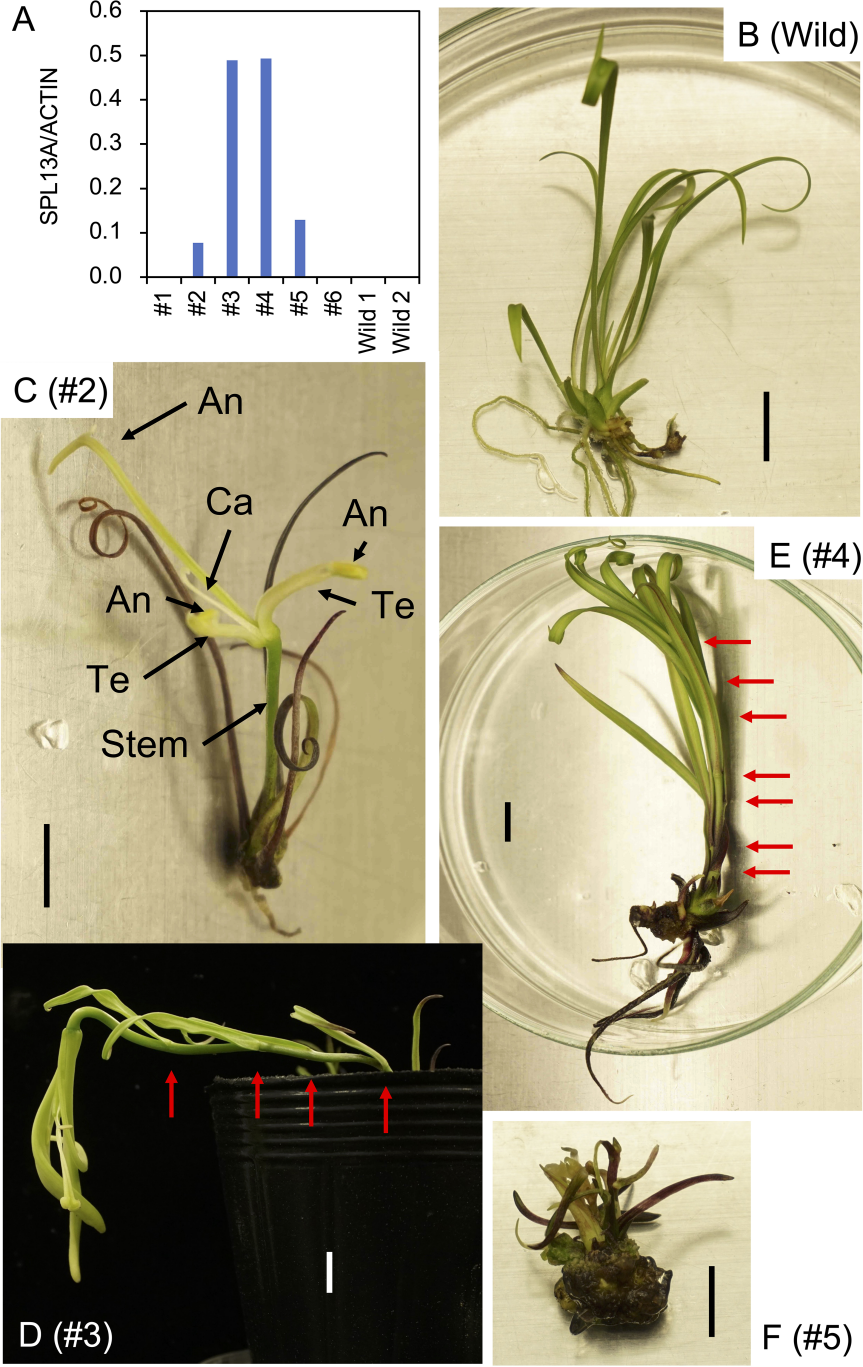
**(A)** *SPL13A* gene expression in leaves of *L. formosanum* plants transformed with a 35S::rSPL13A construct (#1–#6) and callus-derived non-transformed *L. formosanum* plants (Wild 1 and Wild 2) *in vitro*. **(B–F)** Appearance of a non-transformed *L. formosanum* plant (Wild, B) and the transformed *L. formosanum* plant #2 **(C)**, #3 **(D)**, #4 **(E)**, and #5 **(F)**. **(D)** Image of a plant that developed a flower bud *in vitro* and was transplanted into a pot. Red arrows on **(D, E)** indicate nodes. Scale bar = 1 cm. An, anther; Ca, carpel; Te, tepal.

To examine the function of SPL13A, we transformed *L. formosanum* plants with a 35Sp::*rSPL13A* construct that expressed miR156-resistant *SPL13* under the control of the 35S promoter. Six independent plants were regenerated from approximately 1000 callus inoculated with *Agrobacterium* and their bulblets were developed *in vitro*. Of the six transgenic lines, four (#2, #3, #4, and #5) exhibited *SPL13A* expression in the leaves of *in vitro* plantlets, whereas the other two (#1 and #6) and two non-transformed wild type plants (Wild) regenerated from calluses showed faint *SPL13A* expression levels (**Figure 2A**). The most typical features observed in the *in vitro* plants were stem elongation and floral organ development (**Table 1**). Non-transformed lily plants grown *in vitro* developed scaly leaves (the basal part of each leaf enlarged to develop a bulb scale) but did not exhibit stem elongation (**Figure 2B**). In line #2, the stem elongated after the development of four leaves, and floral organs, including two tepals, three anthers, and one carpel, were found (**Figure 2C**). Line #3 had an elongated stem and developed flower buds *in vitro*. As the shoot reached the top of the culture vessel, this plant was transferred to a pot filled with soil in order to allow further growth (**Figure 2D**). The plant flowered after developing four stem leaves (nodes), and the flower organs included four tepals, three stamens, and one pistil (**Supplementary Figure S1**). Line #4 had an elongated stem on a small bulblet *in vitro* (**Figure 2E**). The stem of line 4 developed many leaves but did not flower *in vitro*. Line #5 produced scaly leaves similar to those of non-transformed plants but sometimes produced short stems *in vitro* (**Figure 2F**), which did not grow further. Lines #1 and #6, which did not express the transgene, showed similar morphology to that of non-transformed plants, and stem elongation was not observed *in vitro* (data not shown). In addition to the abovementioned morphogenetic changes, anthocyanin pigmentation was observed in the bulb scales, leaves, and roots of lines #2, #3, #4, and #5, whereas non-transformed plants showed weak anthocyanin pigmentation (**Figure 2**; **Supplementary Figure S2**).

**Table 1:** Phenotypic features observed in the transformed *Lilium formosanum* plants*.

	Stem elongation		Floral organ development		Anthocyanin accumulation	Altered leaf angle**
	*in vitro*	in pot	*in vitro*	in pot		
#2	Yes	No	Yes	No	Yes	Yes
#3	Yes	Yes	Yes	No	Yes	Yes
#4	Yes	Yes	No	Yes	Yes	Yes
#5	Yes	No	No	No	Yes	Yes

*Four regenerated plants (#2–#5) exhibiting the rSPL13A transgene expression are shown.

**Scaly leaves and stem leaves extended upward.

**Figure 3:**
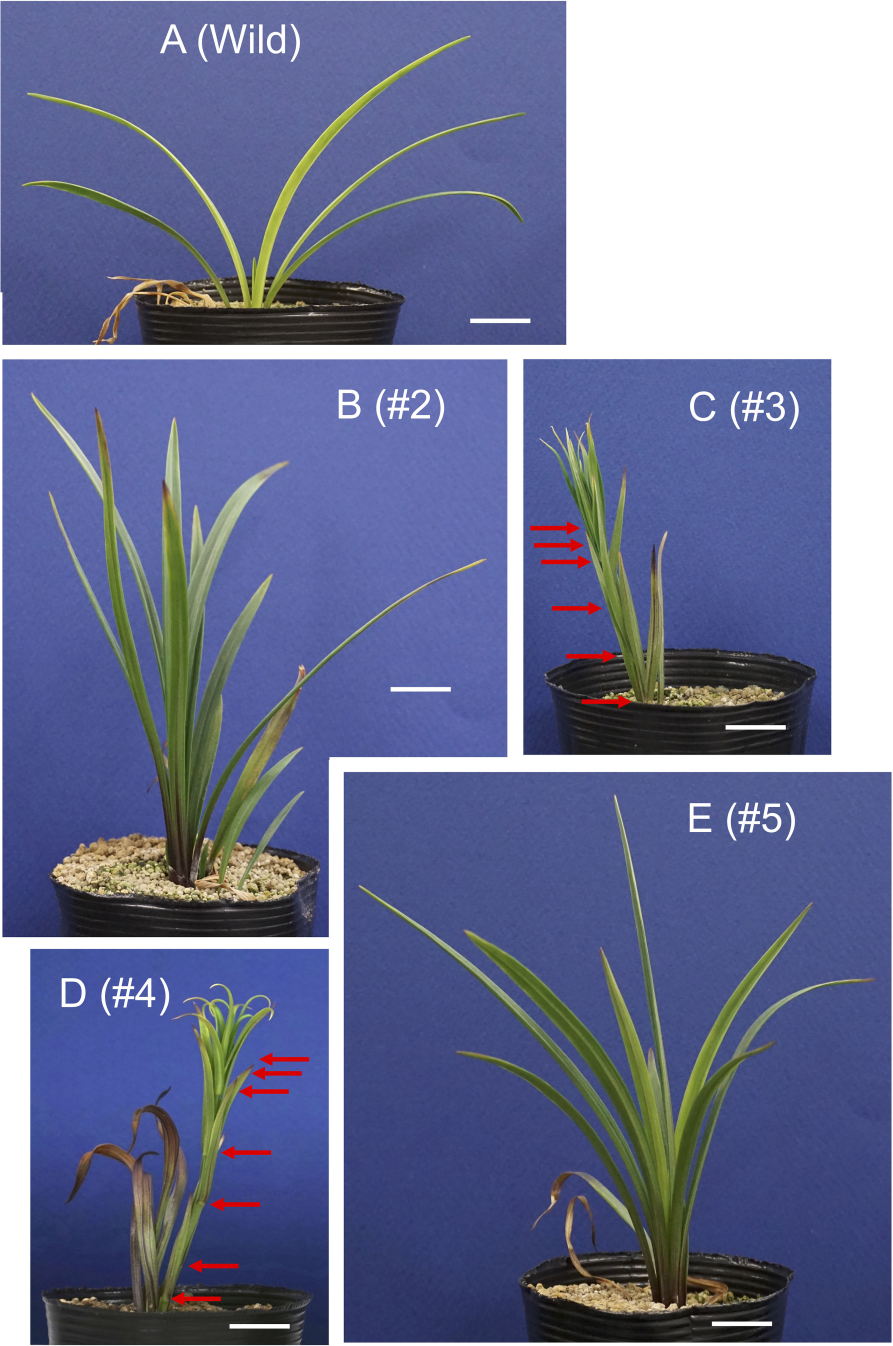
Shoot appearance of the *L. formosanum* plants transformed with a 35S::rSPL13A construct in pots. **(A)** Callus-derived, non-transformed *L. formosanum* plants (Wild). **(B–E)** transformed plant #2 **(B)**, #3 **(C)**, #4 **(D)**, and #5 **(E)**. Bulblets developed *in vitro* were cultivated in soil for 4 weeks without cold exposure prior to planting. Red arrows on **(C, D)** indicate nodes. Scale bar = 2 cm.

After the propagation of transgenic plants *in vitro*, three bulblets of four transformed plant lines (#2, #3, #4, and #5) and non-transformed bulblets were transplanted into pots and cultivated in a growth chamber. Shoot appearance in representative plants 4 weeks after transplanting is shown in **Figure 3**. Scaly leaves extended transversely in the non-transformed plants regenerated from calli (**Figure 3A**). In contrast, scaly leaves extended upwards in lines #2 and #5 (**Figure 3B, E**). All three plants in line #3 and two of the three plants in line #4 had elongated stems after the development of four to seven scaly leaves that extended upwards (**Figures 3C, D**, **Table 1**; **Supplementary Figure S3**).

Generally, *L. formosanum* accumulates anthocyanins near the base of its scaly leaves. Anthocyanin coloration was deeper in transformed plants than in non-transformed plants (**Supplementary Figure S4**). In addition, anthocyanins accumulated along the midrib on the abaxial side of the scaly leaves in the transformed plants, which was not observed in non-transformed plants. The top half of the scaly leaves was also deeply pigmented in line #4 (**Supplementary Figure S3**).

**Figure 4:**
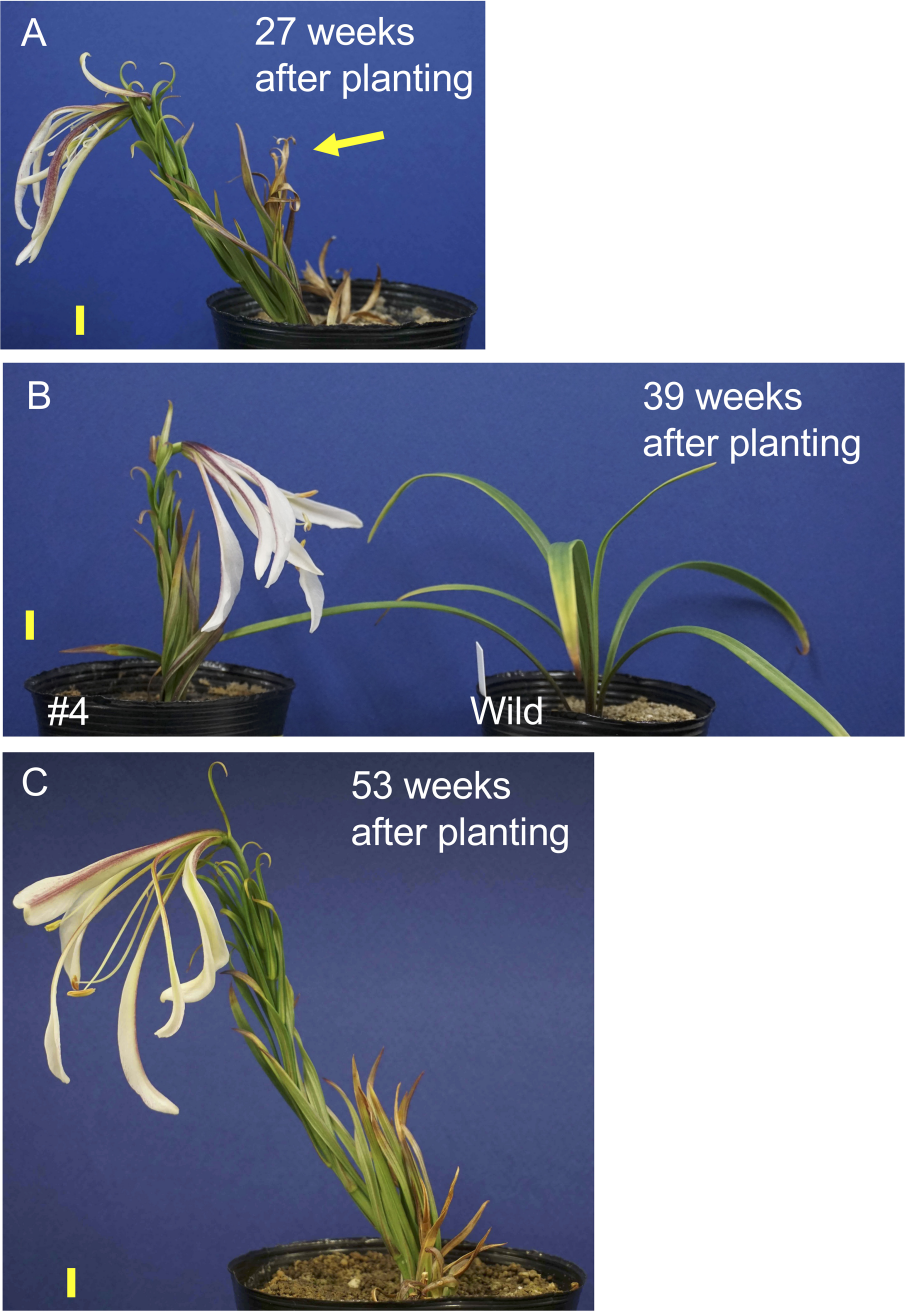
Shoot appearance of the transformed *L. formosanum* line #4 plant at 27 **(A)**, 39 **(B)**, and 53 weeks **(C)** after planting in a pot. The yellow arrow in the panel A indicates 1st shoot, which was withered. A callus-derived non-transformed plant (Wild) is shown in **(B)** Note that these plants were not subject to chilling. Scale bar = 1 cm.

One transgenic plant in line #4 exhibited flowering, whereas other plants in this line did not flower in the pots. After the first sprouted shoot withered, a new shoot appeared and flowered 27 weeks after planting (**Figure 4A**). Similarly, after the second shoot withered, a third shoot sprouted and flowered 39 weeks after planting (**Figure 4B**). After the third shoot withered, a fourth shoot emerged and flowered 53 weeks after planting (**Figure 4C**). Note that all transgenic plants were grown at a chamber maintained at 25°C and chilling treatment was not applied during the culture. Under these conditions the non-transformed plants never exhibited stem elongation (**Figure 4B**). When the bulblets were treated with cold, non-transformed plants exhibited elongated stems and flowered in pots (**Supplementary Figure S5**). Internode length in line #4 (17.5 cm height, 28 leaves, 0.63 cm internode length, **Figure 4C**) was shorter than that in a non-transformed plant (39 cm height, 39 leaves, 1.0 cm internode length, **Supplementary Figure S5**).

Leaves emerging from elongated stems extended upward, parallel to the stems, and the lower half attached to the stems in plants in lines #3 and #4 (**Figures 3**, **4**; **Supplementary Figure S3**), whereas those in the non-transformed plants extended diagonally upward from the stem and did not attach to the stem (**Supplementary Figure S5**). Flower appearances were very different: Although *Lilium* species have choripetalous corollas, the lower half of the inner tepals and that of the outer tepals of wild-type *L. formosanum* connected in a manner similar to a mortise-tenon joint, and thus, its flowers looked like gamopetalous corollas (**Supplementary Figure S5**). In contrast, in plants from line #4, the inner and outer tepals were separate and unconnected. In addition, the abaxial side of the outer tepals was anthocyanin-pigmented in line #4 plants, whereas it was white in the non-transformed plants.

**Figure 5:**
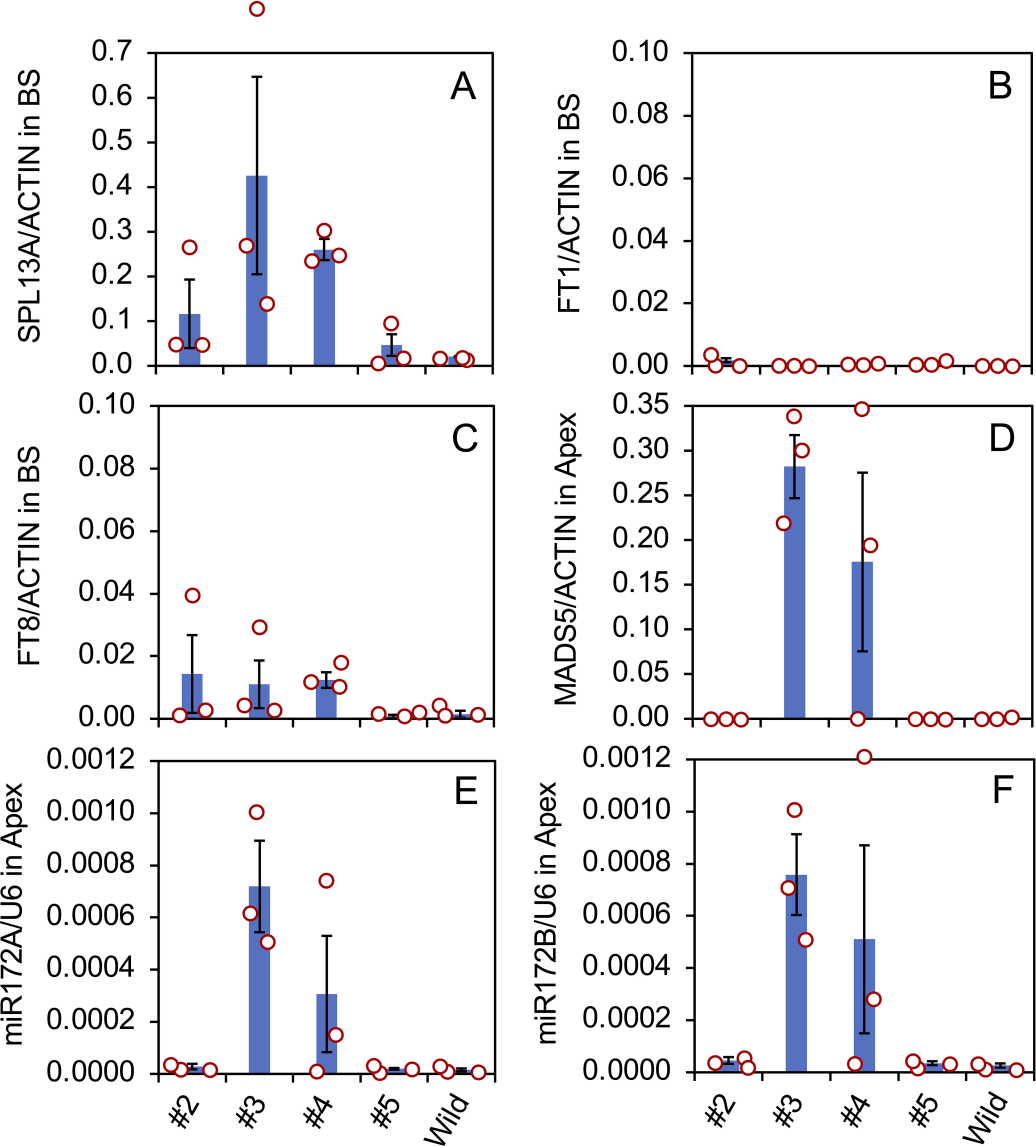
Expression of *SPL13A* **(A)**, *FT1* **(B)**, *FT8* **(C)** in bulbs scale (BS), and *MADS5* **(D)** in shoot apices (Apex), and the accumulation of mature miR172A **(E)** and miR172B **(F)** in shoot apices in *L. formosanum* plants transformed with *rSPL13A* (lines #2–#5) and in non-transformed *L. formosanum* plants (Wild). Vertical bars show the standard error of the means of three plants. Each dot indicates the value of each plant.

Gene expression in the bulb scales and shoot apices of the transgenic plants was evaluated (**Figure 5**) using three vegetatively propagated plants in each transformed line (#2, #3, #4, and #5) and the non-transformed plants (Wild). *SPL13A* was only slightly expressed in the non-transformed plants, indicating that the expression of endogenous *SPL13A* gene is not high in the bulb scale. *SPL13A* was expressed in lines #2, #3, #4, and #5. Lines #3 and #4, which had elongated stems in pots, exhibited relatively high transgene expression. In line #5, one of three plants, shown in **Figure 3**, exhibited transgene expression, whereas the other two plants did not (**Figure 5A**).

The expression of *FT1* in bulb scales was low in all transformed lines and non-transformed plants (**Figure 5B**), but that of *FT8* was relatively high in lines #2, #3, and #4 (**Figure 5C**), suggesting that *FT8* expression was stimulated by *SPL13A* but *FT1* expression was not. The expression levels of lily *MADS5*, which has a function similar to that of *AP1*/*SQUA* (Chen et al., 2008), were high in the shoot apices of lines #3 and #4 (**Figure 5D**). Of the three plants in line #4, two expressed *MADS5* but one without stem elongation did not, indicating that *MADS5* expression correlates strongly with stem elongation.

SPLs positively regulate the expression of *primary-MIR172*, which stimulates floral transition in other species (Wu et al., 2009; Wang, 2014). Accumulation of mature miR172 was evaluated because the *primary MIR172* sequences are not known in *Lilium*. Two mature miR172 sequences were found in lilies, miR172A, 5′-AGAAUCUUGAUGAUGCUGCA*A-*3′, and miR172B, 5′-AGAAUCUUGAUGAUGCUGCA*U-*3′, in which one nucleotide at the 3’ terminal differed (indicated in italic). The accumulation levels of both miR172A (**Figure 5E**) and miR172B (**Figure 5F**) were high in lines 3 and 4. Similar to *MADS5* gene expression, this accumulation was observed in two of the three plants in line #4 but not in the plant without stem elongation.

### 3.2 Gene expression and mature microRNA accumulation in *L. longiflorum*

**Figure 6:**
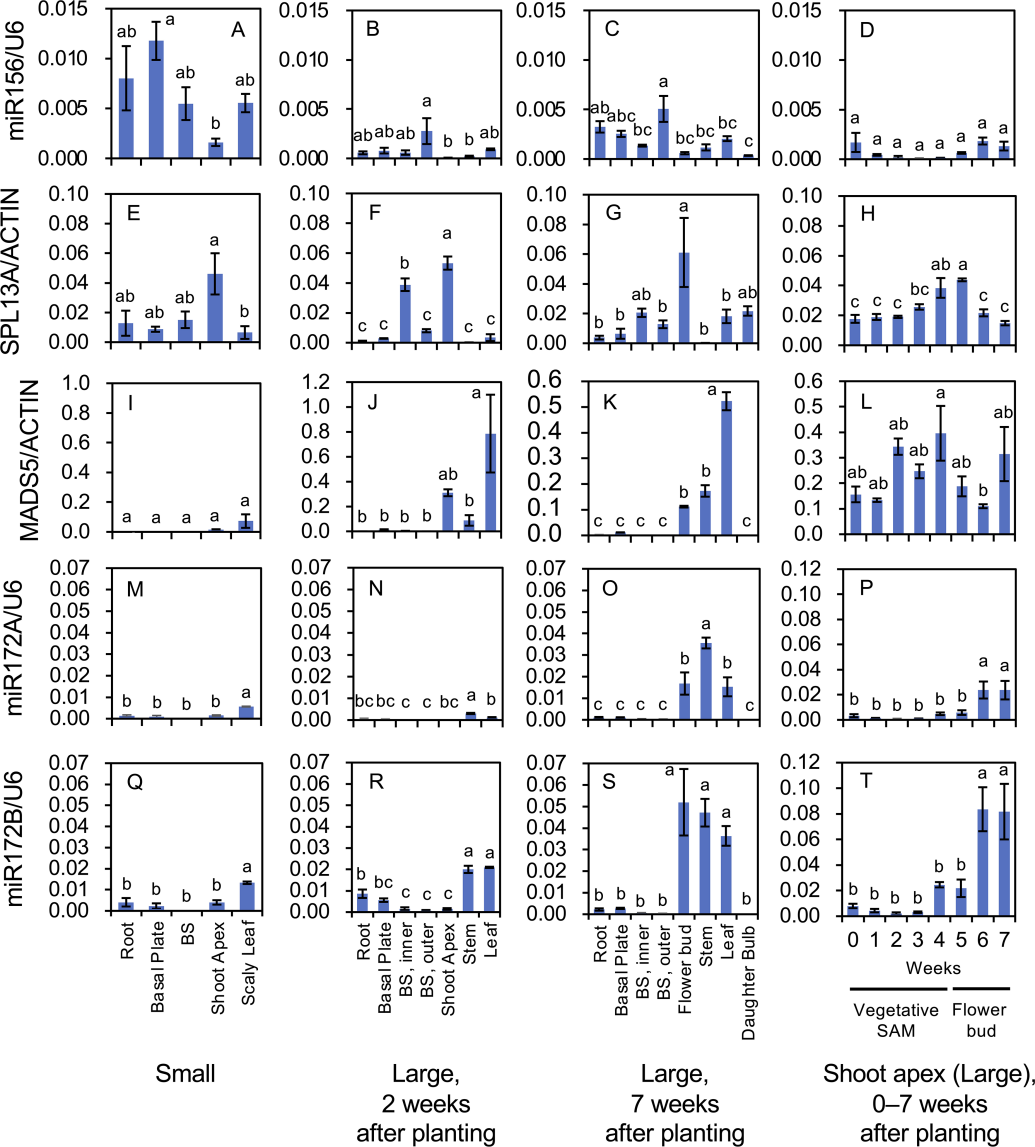
Mature microRNA accumulation of miR156 **(A–D)**, miR172A **(M–P)**, and miR172B **(Q–T)**, and transcript accumulation of *SPL13A* **(E–H)** and *MADS5* **(I–L)** in *L. longiflorum*. **(A, E, I, M, Q)** Accumulation in plants with small bulbs (Small) producing scaly leaves. **(B, F, J, N, R)** Accumulation in plants with large bulbs (Large) 2 weeks after planting. **(C, G, K, O, and S)** Accumulation in the plants with large bulbs (Large) 7 weeks after planting. **(D, H, L, P, T)** Accumulation in shoot apices of the plants with large bulbs 0 to 7 weeks after planting. In plants with large bulbs, stem elongation began soon after planting and tiny flower buds were observed at shoot apices 5, 6, and 7 weeks after planting. Before planting, plants with large bulbs were subject to chilling, while plants with small bulbs were not. Vertical bars show the standard error of the means of three biological replicates. Columns bearing the same letters indicate no significant difference (p < 0.05; Tukey’s HSD test). BS, bulb scale.

**Figure 7:**
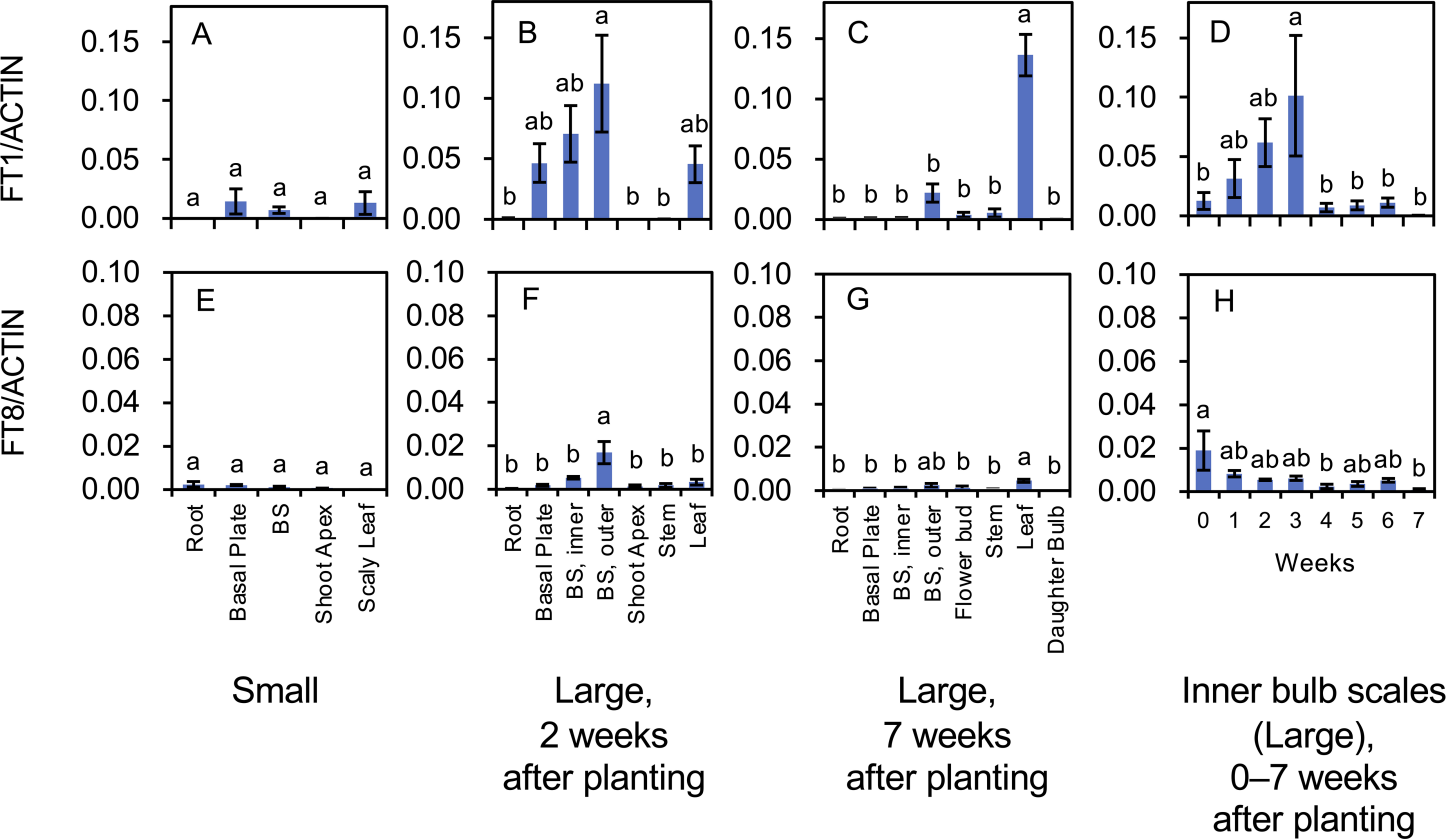
Transcript accumulation of *FT1* **(A–D)** and *FT8* **(E–H)** in *L. longiflorum*. **(A, E)** Accumulation in plants with small bulbs (Small) producing scaly leaves. **(B, F)** Accumulation in plants with large bulbs (Large) 2 weeks after planting. **(C, G)** Accumulation in the plants with large bulbs 7 weeks after planting. **(D, H)** Accumulation in inner bulb scales of the plants with large bulbs 0 to 7 weeks after planting. The plants with large bulbs exhibited elongated stems. Before planting, plants with large bulbs were subject to chilling, while plants with small bulbs were not. Vertical bars show the standard error of the means of three biological replicates. Columns bearing the same letters indicate no significant difference (p < 0.05; Tukey’s HSD test). BS, bulb scale.

The above experiment using *rSPL13*-overexpressing lily plants implied that the miR156/SPL13A module was related to the transition from the juvenile to adult vegetative phase, stem elongation, and flower induction. To explore the relationships between the function and expression profile of the lily *SPL13A*, the spatial and temporal accumulation of mRNA transcripts and mature miRNA was further evaluated using *L. longiflorum ‘*White Heaven.’ First, organ specificity was evaluated in plants with small or large bulbs (**Figures 6**, **7**). Plants with small bulbs (< 1 cm in diameter) derived from scaly propagation developed scaly leaves and roots without stem elongation. Plants with large bulbs (> 7 cm in diameter) exhibited stem elongation and flower induction. As tiny flower buds were observed under a microscope 5 weeks after planting, the plants with large bulbs at 5, 6, and 7 weeks after planting were estimated to be in the reproductive phase.

In plants with small bulbs, high miR156 accumulation was found in the roots, basal plates, bulb scales, and leaves, whereas the accumulation in shoot apices was relatively low (**Figure 6A**). In plants with large bulbs at 2 and 7 weeks after planting, the accumulation of miR156 was relatively high in the outer bulb scales, but was generally low in other organs (**Figures 6B, C**). In the shoot apices of plants with large bulbs, miR156 levels from 0 to 7 weeks after planting (temporal changes) were low, particularly at 2, 3, and 4 weeks after planting (**Figure 6D**).

*SPL13A* transcripts accumulated in the shoot apices of plants with small bulbs (**Figure 6E**), in the inner bulb scales and shoot apices of plants with large bulbs 2 weeks after planting (**Figure 6F**), and in young flower buds of plants with large bulbs 7 weeks after planting (**Figure 6G**), indicating that *SPL13A* transcripts mainly accumulated in the shoot apices regardless of age. From 0 to 7 weeks after planting (temporal changes), *SPL13A* transcripts accumulated at all times in the shoot apices and peaked at 4 and 5 weeks after planting, when the transition from vegetative shoot apices to the inflorescence/floral meristem occurred (**Figure 6H**).

*MADS5* expression in plants with small bulbs was low in all organs, including the shoot apices (**Figure 6I**). *MADS5* expression was mainly observed in shoot apices, stems, and leaves of plants with large bulbs (**Figures 6J, K**). In the shoot apices (temporal changes), its expression peaked around 4 weeks after planting, decreased thereafter, and increased again 7 weeks after planting (**Figure 6L**), consistent with a previous report indicating that *MADS5* expression was detected in the vegetative stems, vegetative leaves, inflorescence meristem, and carpels of young floral buds (Chen et al., 2008).

The accumulation levels of mature miR172B were two- to twenty-times higher than those of mature miR172A in all organs, whereas their accumulation profiles were similar (**Figures 6M–T**). Both miR172A and miR172B accumulated in the scaly leaves of plants with small bulbs (**Figures 6M, Q**). In plants with large bulbs, miR172A and miR172B accumulated in stems and leaves 2 weeks after planting (**Figures 6N, R**) and in flower buds, stems, and leaves 7 weeks after planting (**Figures 6O, S**). In shoot apices (temporal changes), their accumulation levels were not high from 0 to 3 weeks after planting, increased at 4 weeks after planting, and were the highest at 6 and 7 weeks after planting (**Figures 6P, T**).

FT1 and FT8 (LlFT) are involved in flower induction in lilies (Leeggangers et al., 2018; Kurokawa et al., 2020). Transcript accumulation of both FT genes was low in plants with small bulbs (chilling was not applied; **Figures 7A, E**). In plants with large bulbs, transcripts of *FT1* were mainly detected in the basal plates, inner bulb scales, outer bulb scales, and leaves 2 weeks after planting (**Figure 7B**), and in the leaves 7 weeks after planting (**Figure 7C**). *FT1* expression in the inner bulb scales of plants with large bulbs (temporal changes) increased from 0 to 3 weeks after planting and rapidly declined thereafter (**Figure 7D**). The increase in *FT1* expression likely preceded the floral transition in the shoot apices. *FT8* transcripts were accumulated in the outer bulb scales 2 weeks after planting (**Figure 7F**) and small levels of them were found in all organs 7 weeks after planting (**Figure 7G**). *FT8* expression in the inner bulb scales was the highest at 0 weeks after planting (the day the bulbs were planted) and decreased thereafter (**Figure 7H**). In Asiatic hybrid lilies, *FT8* expression levels increase during the cold exposure of bulbs, peak at the end of the cold treatment (the day the bulbs are planted), and rapidly decline thereafter in the bulb scales (Kurokawa et al., 2020).

**Figure 8:**
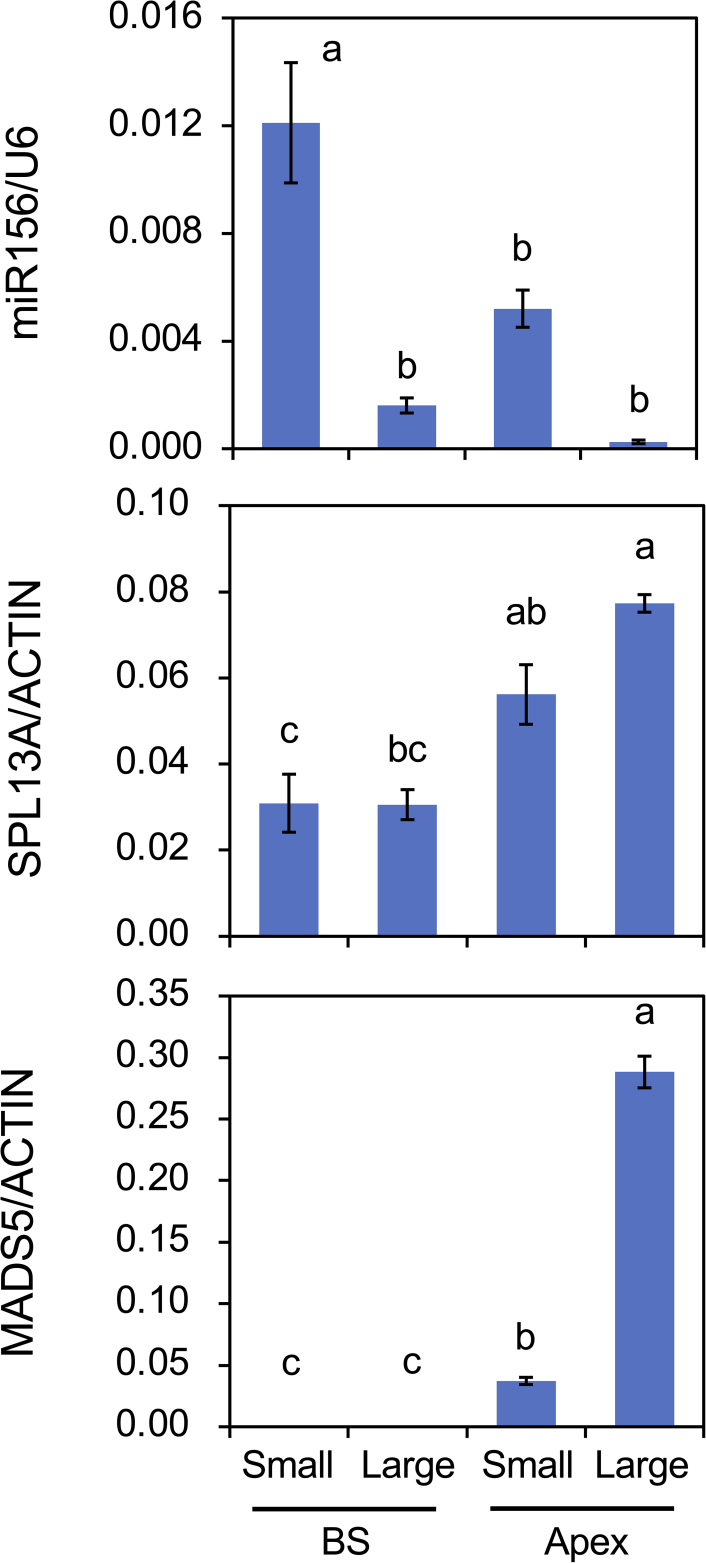
Mature miR156 accumulation and transcript accumulation of *SPL13A* and *MADS5* in bulb scales (BS) and shoot apices (Apex) of plants with small bulbs (Small) or plants with large bulbs (Large). *Lilium longiflorum* was used and the small bulbs were derived from scale-propagation. After cold exposure for 16 weeks, these plants were grown for 2 weeks. Vertical bars show the standard error of the means of five (Small) or three (Large) biological replicates. Columns bearing the same letters indicate no significant difference (p < 0.05; Tukey’s HSD test).

The effect of plant size (age) on the accumulation of mRNA transcripts and mature miRNAs was also evaluated (**Figure 8**). Following cold exposure, small bulbs derived from scaly propagation and large bulbs were planted and cultivated simultaneously for 2 weeks. Plants with small bulbs only developed scaly leaves. miR156 accumulation in the bulb scales and shoot apices was higher in plants with small bulbs than in those with large bulbs. miR156 accumulation in shoot apices was lower than that in the bulb scale of the plants with small bulbs, but miR156 accumulation in shoot apices of plants with small bulbs was significantly detected but that in shoot apices of plants with large bulbs was faint. The accumulation of *SPL13A* transcripts was higher in shoot apices than in bulb scales; however, the difference between plants with small and large bulbs was unclear. In contrast, transcript accumulation of *MADS5* in shoot apices was higher in plants with large bulbs than in those with small bulbs.

**Figure 9:**
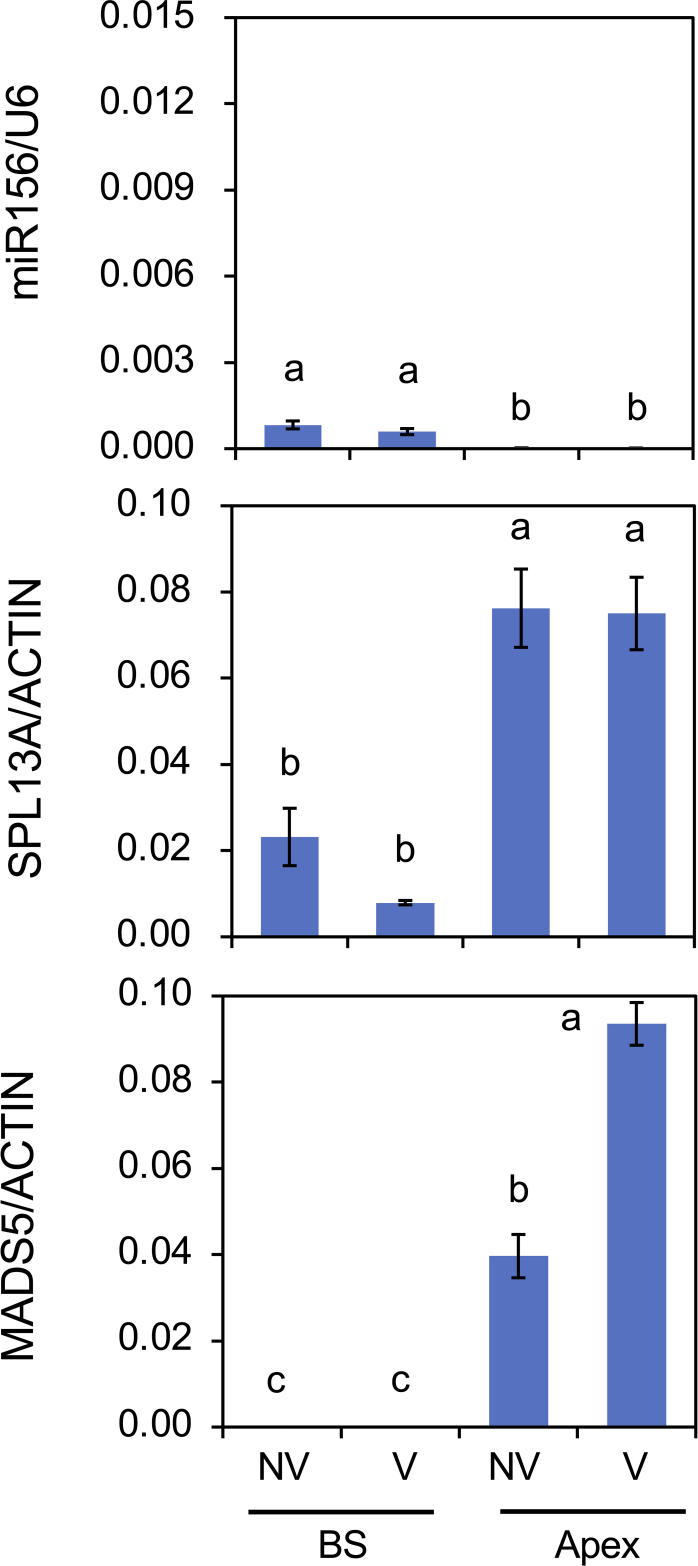
Effects of chilling (vernalization) on mature miR156 accumulation and transcript accumulation of *SPL13A* and *MADS5* in bulb scales (BS) and shoot apices (Apex). Large bulbs of *L. longiflorum* were incubated at 25°C (non-vernalization, NV) or 4°C (V) for 8 weeks and subsequently cultured in a greenhouse for 4 weeks. Vertical bars show the standard error of the means of three biological replicates. Columns bearing the same letters indicate no significant difference (p < 0.05; Tukey’s HSD test).

As winter cold (vernalization) has the strongest effect on lily growth and flowering, the effects of cold exposure at 4°C for 8 weeks on gene expression were examined in plants with large bulbs (**Figure 9**). The amount of miR156 was low at the bulb scale and only faint in the shoot apices. These levels were not influenced by cold exposure. The accumulation levels of *SPL13A* transcripts were higher in shoot apices than in bulb scales, and these were not affected by cold exposure, whereas those of *MADS5* transcripts were increased by cold exposure in shoot apices, indicating that cold exposure stimulated *MADS5* expression but did not influence *SPL13A* expression.

### 3.3 SPL13A was a direct target of miR156

**Figure 10:**
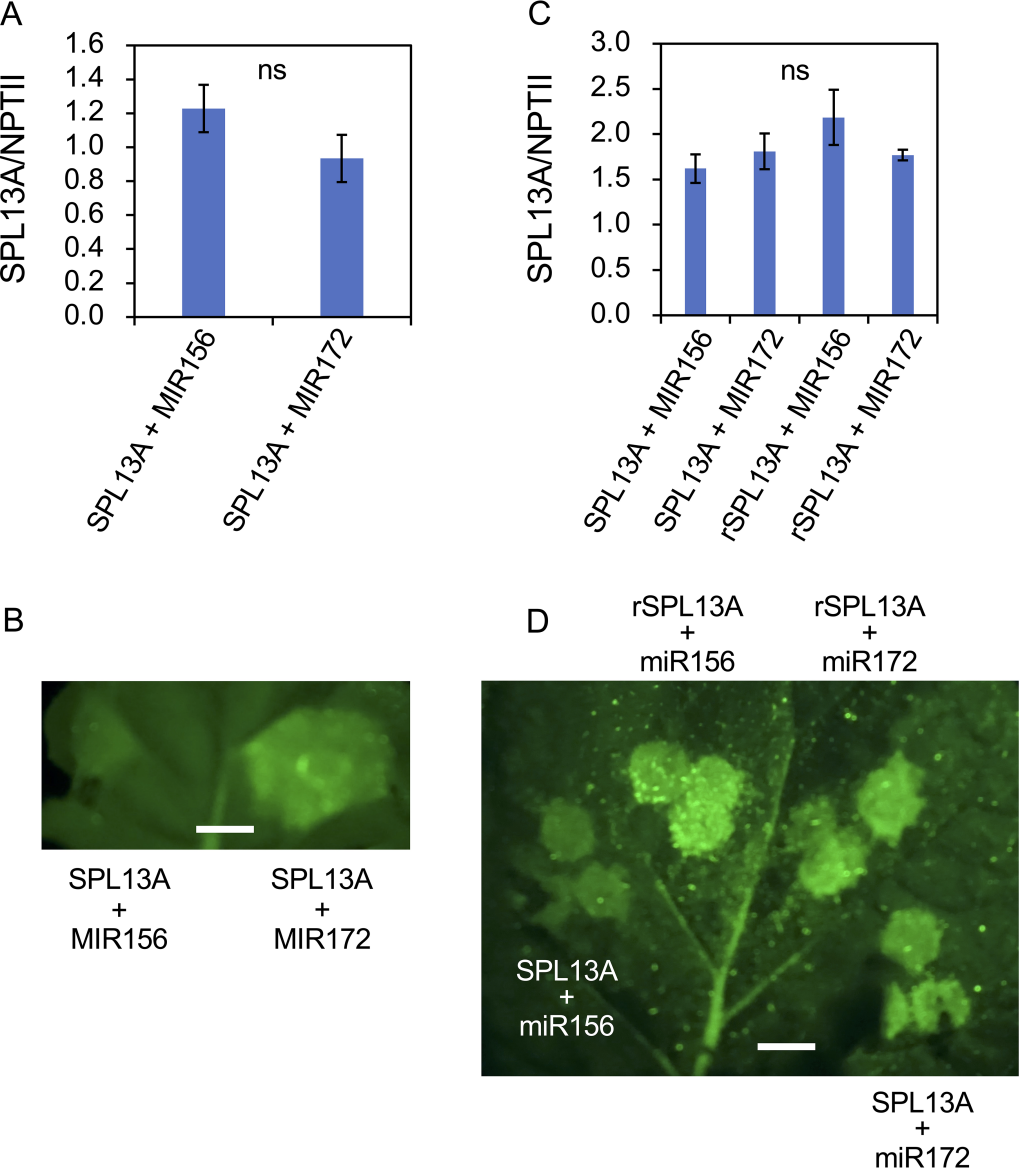
GFP reporter assay to confirm the effects of miR156 on the *SPL13A* target gene. *SPL13A-GFP* **(A, B)** and *SPL13A-GFP* or *rSPL13A-GFP* **(C, D)** were introduced into *Nicotiana benthamiana* leaves together with an *AtMIR156B* or *AtMIR172B* (control) using agroinfiltration. **(A, C)** Transcript accumulation of *SPL13A*. *NPTII* gene on the same binary vector was uses to normalize gene expression. Vertical bars show standard error of the means of four leaves. ns, No significant difference after *t*-test **(A)** and Tukey’s HSD test **(C)**. **(B, D)** GFP fluorescence signals on *N. benthamiana* leaf. White bar = 4 mm.

Although lily *SPL13A* contained a miR156 complementary sequence (**Figure 1**), *SPL13A* transcript accumulation in the bulb scales or shoot apices of plants with large bulbs was similar to that in plants with small bulbs, although the latter accumulated higher levels of miR156 than the former (**Figure 8**). Thus, the effects of miR156 on the *SPL13A* target gene were further evaluated using agroinfiltration into *N. benthamiana* leaves. A binary vector harboring *SPL13A*-*GFP* was introduced into *N. benthamiana* leaves, together with a binary vector containing *AtMIR156B* or *AtMIR172B* (control, **Figures 10A, B**). Transcript accumulation of *SPL13A* was similar between leaves treated with *SPL13A* and *AtMIR172B* (control) and those treated with *SPL13A* and *AtMIR156B* (**Figure 10A**), but GFP fluorescence signals were significantly attenuated when *SPL13A-GFP* was co-infiltrated with *AtMIR156B* (**Figure 10B**). To further confirm that the signal attenuation was correlated with miR156 complementary sequence, *rSPL13A*-*GFP* was introduced instead of *SPL13A*-*GFP*. Although GFP fluorescence signals were attenuated when *SPL13A-GFP* was co-infiltrated with *AtMIR156B*, intensities of GFP signals were similar between leaves treated with *rSPL13A* and *AtMIR172B* (control) and those treated with *rSPL13A* and *AtMIR156B* (**Figure 10D**). Transcript accumulation of *SPL13A* was similar among the leaves treated with the four vector combinations (**Figure 10C**). These results indicate that miR156 represses SPL13A expression mainly at the translational level and that the miR156 binding site is necessary for post-transcriptional repression.

## 4 Discussion

### 4.1 *rSPL13A* overexpression induces stem elongation and flowering under non-inductive conditions

Transgenic research, including gene knockdown and overexpression studies, has played a key role in exploiting and understanding the biological functions of genes that are unique to a species. In this study, the functions of *SPL13A* were evaluated using miR156-resistant *SPL13A* (*rSPL13A*) overexpression. Stem elongation and flowering were induced in the transformed lily lines *in vitro* and in pots. Stem elongation was found in lines #2, #3, #4, and #5 *in vitro*, and in lines #3 and #4 in pots, and flowering occurred in lines #2 and #3 *in vitro* and in line #4 in pots. These four transgenic lines exhibited ectopic expression of *rSPL13A*; in particular, the expression levels of *rSPL13A* in lines #3 and #4 were higher than those in the other lines. A greater number of lines had elongated stems and flowered *in vitro* than in pots, suggesting that the effects of the transgene should appear more easily *in vitro* than in pots, which could be affected by different conditions, such as high sugar supply in our *in vitro* culture. In pots, stem elongation was found in line #3 and stem elongation and flowering were observed in line #4, whereas lines #2 and #5 exhibited an upright appearance of scaly leaves but no stem elongation. These phenotypic changes were correlated with the expression levels of the transgene, which were higher in lines #3 and #4 than in lines #2 and #5.

*Lilium formosanum* (native to Taiwan Island) and *L. longiflorum* (native to the Ryukyu Archipelago) are closely related species belonging to the section Leucolorion b, and *L.* × *formolongi* is an interspecific hybrid. Although several years are usually required from sowing to flowering in many lilies, *L.* × *formolongi* and *L. formosanum* can flower approximately 10 months after sowing (Watanabe, 1993; Mojtahedi et al., 2013). Chilling (vernalization) is not required to induce flowering in seed-derived plants. However, chilling is necessary for bulb-derived plants to induce flowering because non-vernalized *L. formosanum* and *L.* × *formolongi* never flower when bulbs are used for cultivation (Watanabe, 1993; Anderson, 2012). In addition, dormancy is often induced in tissue culture-derived lily bulbs during *in vitro* culture, and cold exposure is usually necessary before planting in the soil to stimulate growth (Yamagishi, 1995; Jásik and de Klerk, 2006). In this study, callus-derived non-transformed *L. formosanum* bulbs without cold exposure developed scaly leaves and exhibited no stem elongation after 12 months of pot culture. Thus, our results show that *rSPL13A* overexpression caused stem elongation and flowering in lily plants under non-inductive conditions.

### 4.2 Timing of vegetative phase change in *Lilium*

Accumulation of miR156 is high in seedlings and decreases with plant growth, and over-accumulation of miR156 delays the timing of vegetative phase change and flowering in many plant species (Chuck et al., 2007a; Wang et al., 2009, 2011; Bergonzi et al., 2013; Zhou et al., 2013; Bhogale et al., 2014). These findings indicate that the roles of miR156 in vegetative phase change are conserved among angiosperms and that the growing stages are in the juvenile or adult phase, which can be defined with the aid of miR156 accumulation levels. This study is the first to evaluate temporal and spatial changes in miR156 accumulation in lilies. miR156 was highly accumulated in plants producing only scaly leaves, whereas its accumulation levels were relatively low in plants with elongated stems, especially at the shoot apices. These observations support the hypothesis stated earlier that scaly leaf-producing lily plants are in the juvenile vegetative phase, and lily plants with elongating stems are in the adult phase.

### 4.3 The miR156/SPL13A module regulates stem elongation and flowering

miR156 negatively regulates *SPL* expression through a combination of transcript cleavage and translational repression or by translational repression (Lei et al., 2016; Xu et al., 2016), and miR156 in Arabidopsis represses *SPL13* expression primarily at the translational step (He et al., 2018). Our transient assay using tobacco plants expressing the *SPL13A-GFP* or *rSPL13A-GFP* fusion protein genes indicates that miR156 in lilies also repressed *SPL13A* expression mainly at the translational level.

High transcript accumulation of *SPL13A* was detected in the shoot apices, where it regulates stem elongation and flower induction, in both juvenile and adult lily plants. Because SPL13A activity was regulated by miR156 mainly at the translational step, its activity levels did not align with the levels of transcript accumulation. In adult lily plants, as miR156 accumulation levels were very low at the shoot apices, we concluded that SPL13A was active enough to induce stem elongation and flower induction. In the shoot apices of juvenile lily plants, the amount of miR156 was relatively low among the five organs but was reliably detected, unlike that in the shoot apices of adult plants, indicating that SPL13A activity should be insufficient to stimulate stem elongation in juvenile plants although *SPL13A* transcripts were accumulated in the shoot apices. We cannot judge whether such a small amount of miR156 completely inhibits SPL13A activity in shoot apices, but such small amounts of miR156 warrant attention because a slight increase in miR156 levels creates a dramatic change in SPL protein amounts, resulting in a significant change in leaf morphology in Arabidopsis (He et al., 2018). Further analysis, including the evaluation of SPL13A protein amounts, will be necessary to confirm that SPL13A activity levels are insufficient to stimulate stem elongation during the juvenile phase.

### 4.4 *SPL13A* stimulates the stem elongation that occurs in the adult vegetative phase

In Arabidopsis and Gramineae species, FT regulates stem elongation by activating or suppressing transcription factors that act on the rib or intercalary meristems (Gómez-Ariza et al., 2019; McKim, 2020). In sugar beets (*Beta vulgaris*), B-BOX TYPE ZINC FINGER 19 acts epistatically over BOLTING TIME CONTROL 1 to promote early bolting and flowering by repressing the downstream bolting repressor BvFT1 and activating the downstream floral activator BvFT2 (Pin et al., 2010, 2012; Dally et al., 2014). Thus, flower induction and stem elongation are regulated by common upstream factors and occur simultaneously in these annual and biennial plant species. However, while the initiation of stem elongation and floral transition occurs at almost the same time in early flowering cultivars of Asiatic hybrid lilies (Kurokawa et al., 2020), stem elongation is followed by floral transition in many lily species and cultivars, including *L. longiflorum* (Roh and Wilkins, 1977; Leeggangers et al., 2018). In this study, stem elongation of *L. longiflorum* plants began soon after planting, and green shoots appeared above the ground between 1 and 2 weeks after planting, whereas floral transition was observed between 4 and 5 weeks after planting. Thus, the initiation of stem elongation is an event that occurs in the adult vegetative phase in most lilies, whereas stem elongation in Arabidopsis, rice, and sugar beets occurs in the reproductive phase. In addition, while chilling is the most effective environmental cue to induce stem elongation and floral transition in lilies, high ambient temperature is exceptionally effective in initiating stem elongation in *L. longiflorum* but has no effect on flower induction (Wang and Roberts, 1970). Stem elongation and flower induction begin at different times in most lilies (Roh and Wilkins, 1977; Leeggangers et al., 2018), and different environmental cues are effective for stem elongation in some *Lilium* species (Wang and Roberts, 1970), indicating that stem elongation and floral transition are regulated independently in lilies and the regulation mechanism to initiate stem elongation in lilies should differ from those in Arabidopsis, rice, and sugar beets. The overexpression of *rSPL13A* stimulated stem elongation under non-inductive conditions, indicating that lily *SPL13A* is involved in the regulatory mechanisms underlying stem elongation. At shoot apices of adult *L. longiflorum* plants, SPL13A was active all the time, from the day when the bulbs are planted, and stem elongation began soon after the planting. Thus, the expression profile of SPL13A supported its function of regulating stem elongation during the adult vegetative phase.

Several studies have reported the involvement of *SPL* genes in stem elongation in other species. As *AP2*-*like* genes suppress the proliferation and elongation of cells in the intercalary meristematic regions of Poaceae (Ji et al., 2019; Patil et al., 2019), *SPLs* indirectly enhance internode elongation through the miR172/*AP2*-*like* gene module (Debernardi et al., 2017; McKim, 2020). However, the miR172/*AP2*-*like* gene module in barley and rice stimulates internode elongation but does not affect the number of nodes (Ji et al., 2019; Patil et al., 2019). In this study, transgenic lines #3 and #4 had elongated stems in pots, and many leaves (nodes) were found on the stems. As these were stem leaves (not scaly leaves), they were newly differentiated during stem development, indicating that SPL13A stimulated both leaf (node) differentiation and internode elongation. Thus, the roles of SPLs in stem elongation differ between lilies and Poaceae.

The internode length of elongated stems in line #4 was shorter than that of non-transformed chilling-treated plants, indicating that although SPL13A stimulates internode elongation, other factors are also necessary for normal internode elongation. As chilling triggers stem elongation, these factors are regulated by dormancy released by chilling. Several factors are involved in stem elongation, some of which are GA-dependent because GA plays a crucial role in internode elongation of Gramineae species (McKim, 2019) and in bud elongation of tree species after breaking dormancy (Singh et al., 2017; Busov, 2019). As anti-GA reagents (e.g., paclobutrazol) shorten stem length in lilies (Jiao et al., 1986, 1991), GA-dependent factors should be involved in the elongation of lily stems. In addition, because glycerol in the bulb scales inhibits sprouting and flowering in *L. longiflorum* (Lazare et al., 2019), nutrient-related signals are thought to stimulate or suppress stem elongation. Because plasmodesmata are closed and symplastic transport of GA and sugars into cells at shoot apices is inhibited in dormant lily bulbs, but winter cold opens plasmodesmata and restores symplastic transport (Pan et al., 2023), it is highly probable that GA-dependent and nutrient-related signals are responsible for stem elongation after release from dormancy.

### 4.5 rSPL13A overexpression altered the angle of leaf blade and stimulated anthocyanin accumulation

*SPL* genes are often involved in morphological changes in adult leaves; for example, in Arabidopsis, these changes include leaf blade outgrowth promotion, petiole development suppression (resulting in an increase in the length-to-width ratio of the leaf blade), trichome production on the abaxial leaf surface, and an increase in the degree of serration at the leaf margin (Hu et al., 2023). As *L. formosanum* has no leaf petiole (leaf blades attach directly to stems), no leaf trichomes, and a smooth leaf margin, morphological changes such as those reported in Arabidopsis were not found in the overexpression lines. Instead, both scaly and stem leaves extended upward in plants overexpressing r*SPL13A*. Because the length and width of scaly leaves were similar between transgenic and non-transgenic lilies (data not shown), rSPL13A overexpression altered the angle between the leaf blade and the stem. However, whether this morphological change is correlated with vegetative phase change is not clear, because changes in leaf angle with lily growth are not found in *L. formosanum*. In addition, the relationship between the leaf angle and vegetative phase change or floral induction has not yet been studied in *Lilium*. LIGULELESS1 (*SPL8*, clade III) in Gramineae species is involved in decreasing the angle of the leaf blade (Liu et al., 2019) but such functions are not known for *SPL* genes targeted by miR156. Further examination is necessary to clarify whether changes in leaf angle are correlated with vegetative phase changes.

Deeper anthocyanin pigmentation was observed in the roots, leaves, and flower tepals of the *rSPL13A* overexpression lines, indicating that lily *SPL13A* stimulates anthocyanin biosynthesis. However, *SPL* often negatively regulates anthocyanin biosynthesis in other plants (Wang et al., 2020). For example, *SPL9* in Arabidopsis (clade VIII) directly inhibits the expression of anthocyanin biosynthetic genes and destabilizes the MYB-bHLH-WD40 transcriptional activation complex (Gou et al., 2011; Cui et al., 2014). VcSPL12 in blueberry (clade V) also suppresses the expression of anthocyanin biosynthesis genes and a *VcMYBPA1* positive regulator gene (Li et al., 2020). Thus, the effects of *SPL13A* in lilies are the opposite of those observed in these species. However, *SPL7* (clade I) in Arabidopsis and *Salvia miltiorrhiza* positively regulates anthocyanin biosynthesis through different genetic cascades (Zhang et al., 2014; Chen et al., 2021). The regulation of anthocyanin biosynthesis by *SPL* genes varies depending on *the SPL* genes and plant species.

### 4.6 SPL13A mainly activates genes involved in the age pathway

Accumulation levels of mature miR172A and miR172B increased at 4 and 5 weeks after planting in the shoot apices of adult *L. longiflorum* plants, suggesting that miR172 is involved in flower induction because floral transition occurs between 4 and 5 weeks after planting. Their accumulation levels further increased 6 and 7 weeks after planting. In addition to the roles of miR172 in floral transition (Ó’Maoiléidigh et al., 2021; Debernardi et al., 2022), miR172 has additional roles in floral organ development and its accumulation levels often increase in inflorescence and flower buds (Chuck et al., 2007b; Debernardi et al., 2017; Gattolin et al., 2020).

*AP1* and *primary MIR172* are the major genes involved in the age pathway that induces flowering. In *rSPL13A*-overexpressing lines, high accumulation of *MADS5* transcripts, mature miR172A, and mature miR172B was observed in stem-elongated plants. Thus, our results indicate that *SPL13A* controls flowering time in lilies by regulating the genes involved in the age pathway.

Lily *FT1* and *FT8* (*LlFT*) are the major factors involved in the vernalization pathway (Leeggangers et al., 2018; Kurokawa et al., 2020). In this study, overexpression of *rSPL13A* did not stimulate *FT1* expression in lily lines, suggesting that *FT1* expression is decoupled from the upregulation of *SPL13A* in lilies, although *SPL* genes in Arabidopsis and wheat indirectly enhance *FT* expression via a miR172/AP2-like module (Mathieu et al., 2009; Debernardi et al., 2022). This observation indicated that stem elongation and flowering in the transgenic lines were not caused by *FT1*. On the contrary, *FT8* expression was stimulated in the bulb scales of *rSPL13A*-overexpressed lines. However, the expression levels in these lines were low. In addition, *FT8* expression levels varied greatly among the plants in lines #2, #3, and #4 and were poorly correlated with the presence or absence of stem elongation (for example, two of the three plants in line #3 exhibited low *FT8* expression, but all three plants had elongated stems). Thus, we believe that the elevated expression of *FT8* found in the *rSPL13A*-overexpressing lines contributed little to stem elongation and flowering.

Chilling stimulates *SPL15* expression but has little effect on *SPL3* expression in Arabidopsis (Deng et al., 2011). In the present study, cold exposure did not affect *SPL13A* expression in lilies. As cold exposure did not induce *SPL13A* expression, and *rSPL13A* overexpression did not stimulate *FT1* expression and only weakly enhanced *FT8* expression, *SPL13A* is poorly involved in the vernalization pathway in lilies.

The expression of *SPL13A* and *MADS5* was high and peaked approximately 4 weeks after planting in the shoot apices of adult *L. longiflorum* plants. The accumulation levels of mature miR172A and miR172B also increased 4 weeks after planting. At the bulb scales of the adult plants, *FT1* expression increased from 0 to 3 weeks after planting. These expression profiles suggest that SPL13A, miR172, FT1, and MADS5 are highly involved in flower induction and that both the age and vernalization pathways are responsible for flower induction in lilies.

## 5 Conclusion

*SPL* genes are involved in phenotypic and physiological changes that occur during the adult vegetative phase in many plant species (Preston and Hileman, 2013; Lawrence et al., 2021). Lily SPL13A can stimulate stem elongation, which is an event that occurs during the adult vegetative phase. Although the regulatory mechanisms underlying stem elongation accompanied by floral induction have been well-evaluated, mainly in Arabidopsis and Poaceae (Gómez-Ariza et al., 2019; McKim, 2020), the mechanisms underlying floral induction-independent stem elongation are not well understood. These results will help understand such mechanisms. SPL13A also stimulated mature miR172 accumulation and *MADS5* expression, indicating that SPL13A regulates the genes involved in the age pathway to induce flowering.

**Figure 11:**
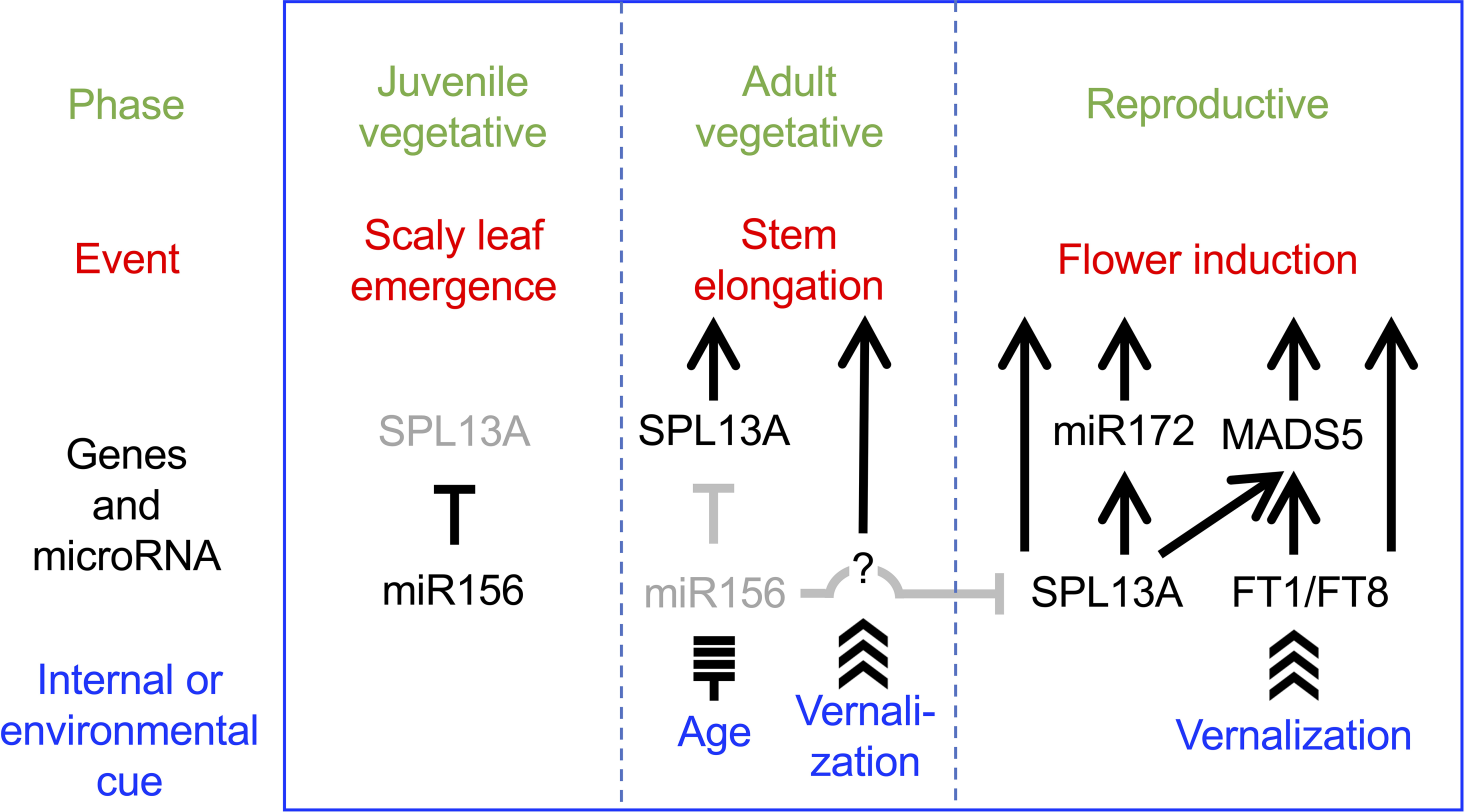
Heuristic model in which SPL13A stimulates stem elongation and flowering in lilies. SPL13A activity is repressed by miR156 in the juvenile vegetative phase. SPL13A is activated as miR156 levels decline in the adult vegetative phase. Then, SPL13A and unknown factors, which should be activated by vernalization, induce stem elongation. SPL13A and FT (FT1 and FT8) are both necessary to induce flowering. Arrows and T-bars indicate positive and negative regulation, respectively, but whether it is a direct or indirect effect is not considered.

The current data suggest a heuristic model in which *SPL13A* is involved in stem elongation and flower induction (**Figure 11**). In the juvenile vegetative phase, miR156 levels are high and *SPL13A* activity is low in wild-type lilies, which produce scaly leaves. As the miR156 levels decrease, *SPL13A* activity increases. If miR156 levels are sufficiently decreased, *SPL13A* stimulates stem elongation at shoot apices. Unlike in Arabidopsis, rice, and sugar beets, *FT* genes are poorly involved in stem elongation in lilies. Unknown vernalization-induced factors may also be involved in stem elongation. At the apex of the elongating stems, conversion from the vegetative shoot apical meristem to the inflorescence meristem and floral meristem occurs, which is stimulated by both the age (SPL13A, miR172, and MADS5) and vernalization (FT1, FT8, and MADS5) pathways underlying flower induction. Further studies are necessary to confirm this heuristic model, including a study to identify the genes regulated by SPL13A and to clarify the functions of other lily *SPL* genes.

## Data Availability

The GenBank/DDBJ/EMBL accession numbers of SPL13A are LC819245 (Asiatic hybrid lily ‘Lollypop’), LC819247 (Oriental hybrid lily ‘Dizzy’), and LC819246 (*L. longiflorum*).
