# Supplementary material for: Overexpression of lily MicroRNA156-resistant SPL13A stimulates stem elongation and flowering in Lilium formosanum under non-inductive (non-chilling) conditions
Source: Front Plant Sci. 2024 Oct 18;15:1456183. doi: 10.3389/fpls.2024.1456183 (PMC11527630; doi:10.3389/fpls.2024.1456183)
Supplement: Supplementary file 1 [file DataSheet1.pdf]

Supplementary TABLE S1. List of PCR primeras

	Forward primer	Reverse primer
Vector construction		
35S::rSPL13A	AAA tctaga AGTCATGGATTGGGATTTGAAG	ACTTAACAAGCTCAATGCGCAATCAGAGTCGTGAACATGGG
	TGCGCATTGAGCTTGTTAAGTTCACCCATCCAATCTTCAAG	GGT gagctc TACTGCCAAGAAAATGGAAGAG
35S::SPL13A(rSPL13A)-GFP	AAA tctaga AGTCATGGATTGGGATTTGAAG	TCCGCCGCTTCCGCCACCCTGCCAAGAAAATGGAAGAG
	GGTGGCGGAAGCGGCGGAGGTAGTGGCGGAGGTAGCATGAGTAAAGGAGAAGAAC	ATT gagctc TTATTTGTATAGTTCATCCATG
35S::AtMIR156B	TTT tctaga TTTGACAAAACATCATGTCTCTCCA	TTC gagctc CTTCAAACTCAAACGCAAAGACTTT
35S::AtMIR172B	ATC tctaga CTAGTCTTGTGTGCACCCATTTATGTG	CTC gagctc CTCAAGTCAAGATCAAAGGCAAAATAG
qRT-PCR		
SPL13A	ACAATCGGCGTCGACGCAAG	TGTGGAAGTGTTGTTGCCTG
FT1	ATGGAGAATAGGAGTGATCCTCTTGCTG	TTCTCTAAGGTTTGGGTTACTTGG
FT8	GAGGGAGTATTTRCACTGGTTGGT	TAAAGATCAGAAAARTTCCGGGTA
MADS5	ACTCCAACAACTAGAGCAGC	AAGCCTCCATCTTCTGGAAC
ACTIN	GGAGTGAGCCACACAGTTCC	ATAGCTCTTCTCCACAGAGG
NPTII	TGAATGAACTGCAGGACGAG	ATACTTTCTCGGCAGGAGCA
Stem-Loop PCR		
miR156, cDNA synthesis		GTTGGCTCTGGTGCAGGGTCCGAGGTATTCGCACCAGAGCCAACGTGCTC
miR156, qRT-PCR	TCGCGTGACAGAAGAGAGT	GTGCAGGGTCCGAGGT
miR172A, cDNA synthesis		GTTGGCTCTGGTGCAGGGTCCGAGGTATTCGCACCAGAGCCAACTTGCAG
miR172A, qRT-PCR	TCGCGAGAATCTTGATGATG	GTGCAGGGTCCGAGGT
miR172B, cDNA synthesis		GTTGGCTCTGGTGCAGGGTCCGAGGTATTCGCACCAGAGCCAACATGCAG
miR172B, qRT-PCR	TCGCGAGAATCTTGATGATG	GTGCAGGGTCCGAGGT
U6, cDNA synthesis		CGATTTGTGCGTGTCATCCTTGC
U6, qRT-PCR	CGGGGACATCCGATAAAATTGGAACG	CGATTTGTGCGTGTCATCCTTGC

Lowercase letters indicate restriction sites. Overlapped regions are shown by underlines.

Supplementary TABLE S2. GenBank/DDBJ/EMBL accession numbers of Arabidopsis SPL sequences

SPL	Accession number
AtSPL13	NP_568731
AtSPL2	NP_199141
AtSPL10	NP_001323272
AtSPL11	NP_001077603
AtSPL8	NP_973738
AtSPL9	AY046007
AtSPL15	NP_191351
AtSPL6	NP_177077
AtSPL3	U50647
AtSPL4	NP_175723
AtSPL5	CAB56772
AtSPL7	OAO96340
AtSPL14	NP_173522
AtSPL16	NP_177784
AtSPL12	NP_191562
AtSPL1	NP_850468

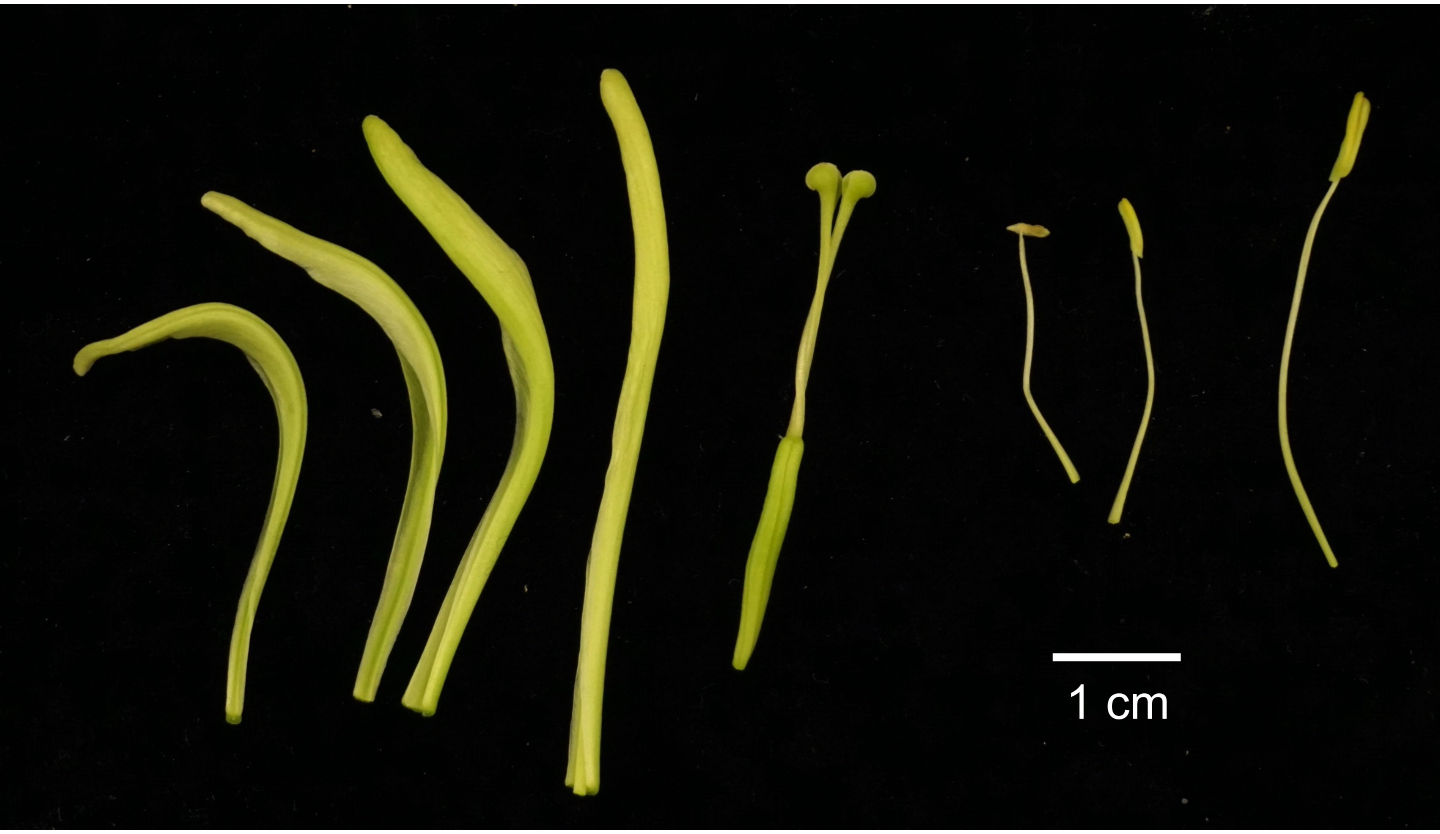

Supplementary Figure S1 The flower of the line #3 transformed plant (Figure 2D) consisted of (from left to right) four tepals, one pistil, and three stamens.

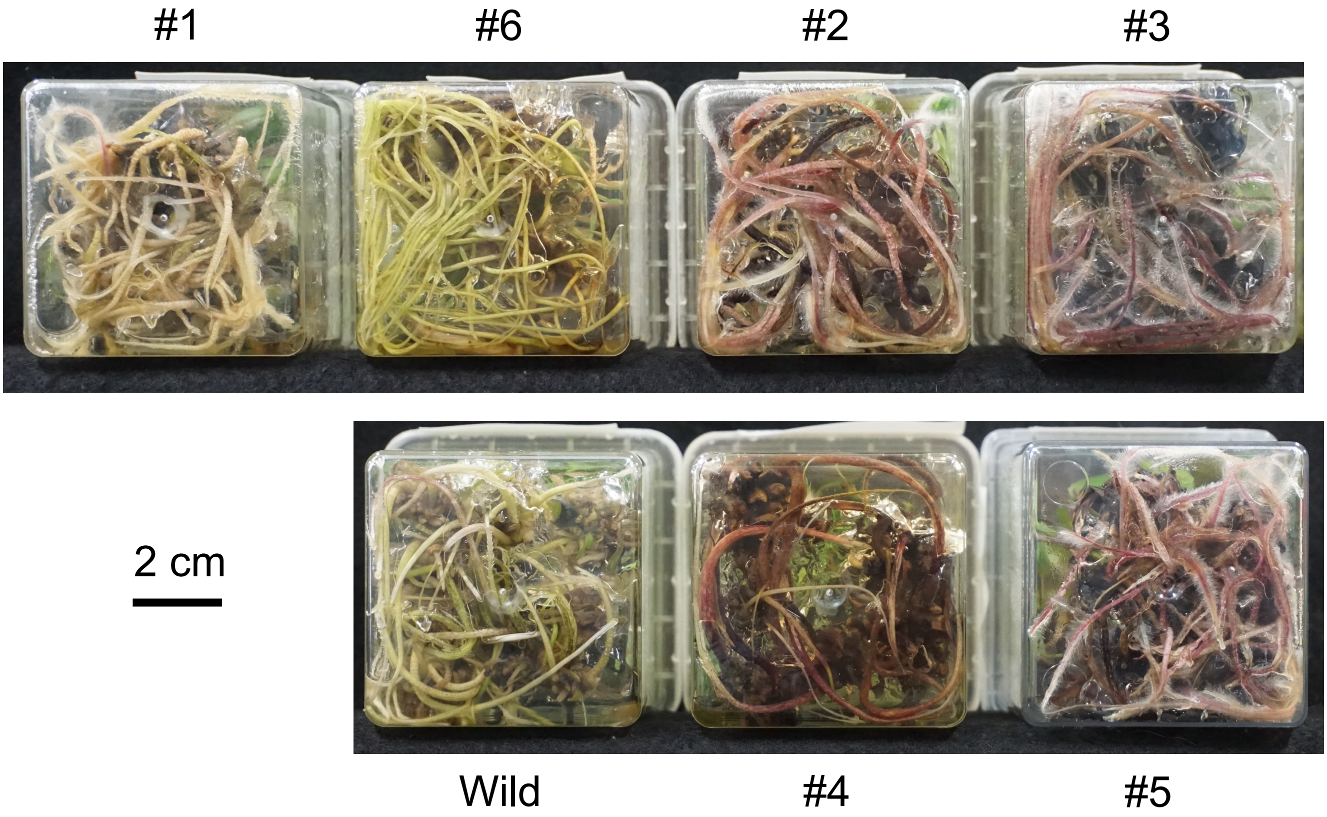

Supplementary Figure S2 Root colors of transformed plants (lines #1–#6) and non-transformed plants (Wild) *in vitro*. Photographs were taken from the bottom of the culture vessels.

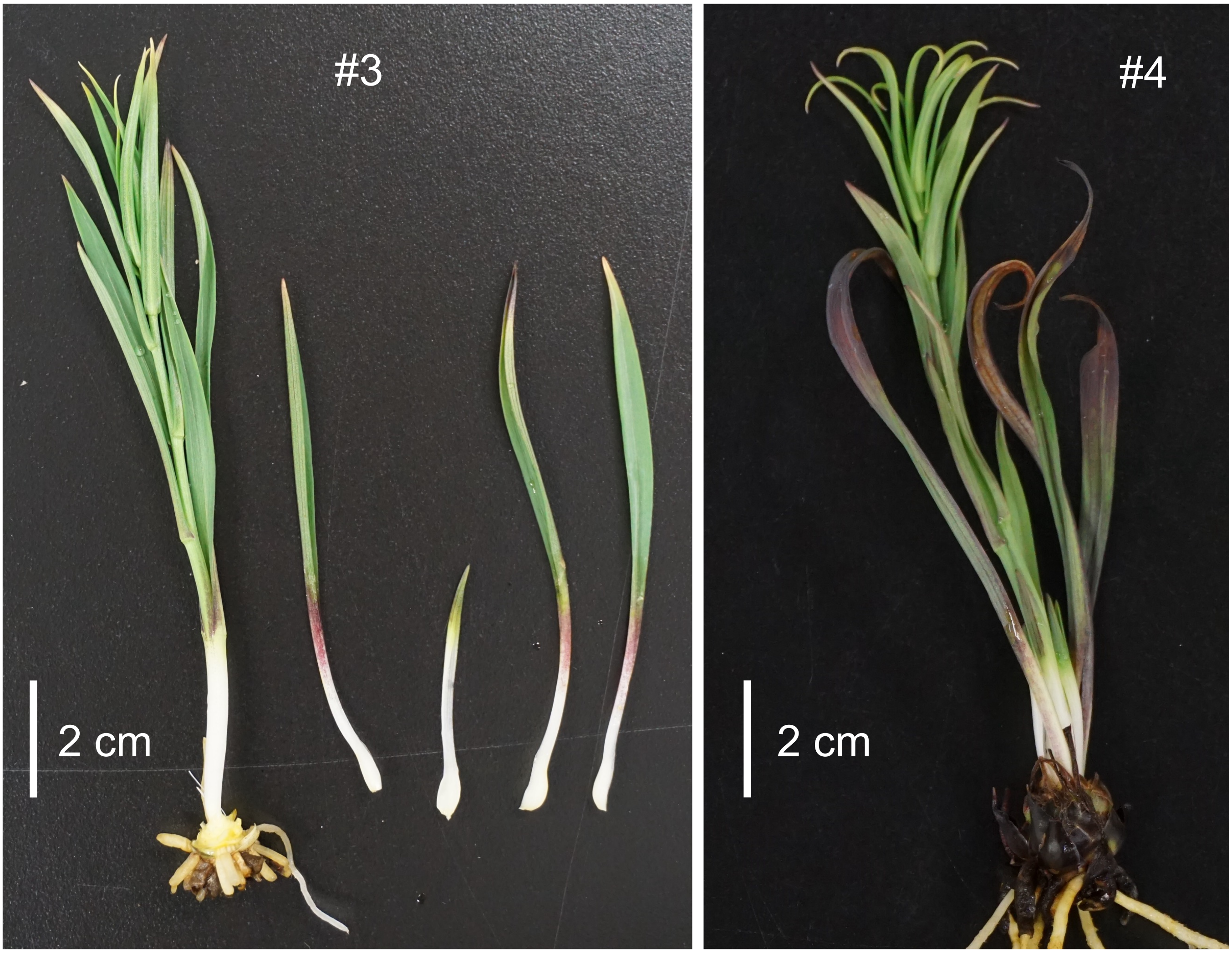

Supplementary Figure S3 The transformed plants of lines #3 and #4, which were taken out of the pots (Figure 3). The line #3 plant consisted of an elongating shoot with 4 scaly leaves, and the line #4 plant consisted of an elongating shoot with 7 scaly leaves. The upper half of scaly leaves were deeply anthocyanin-pigmented in the line #4 plant.

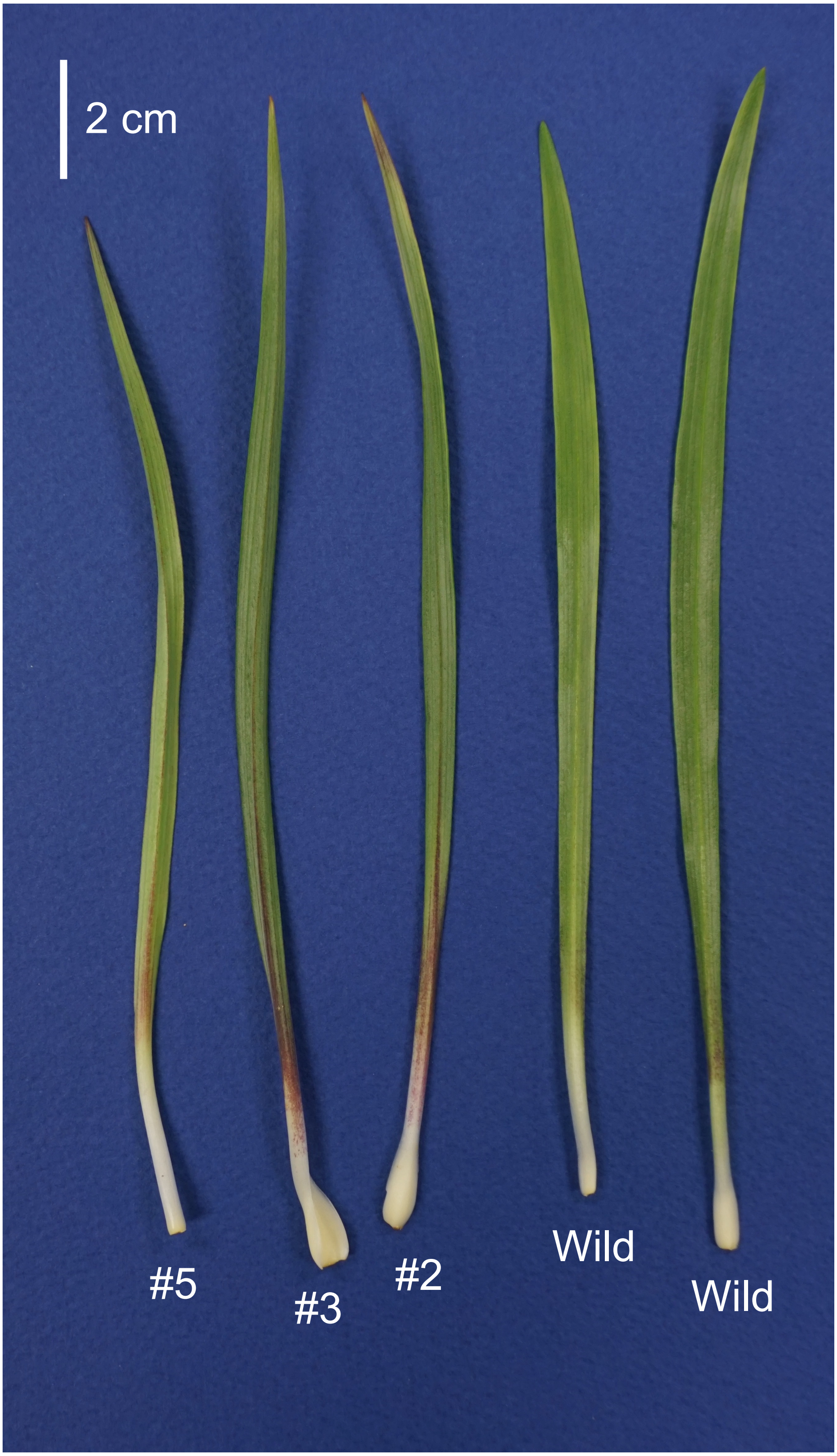

Supplementary Figure S4 Abaxial side of scaly leaves of the transformed plants from lines #5, #3, and #2, and of two non-transformed plants (Wild).

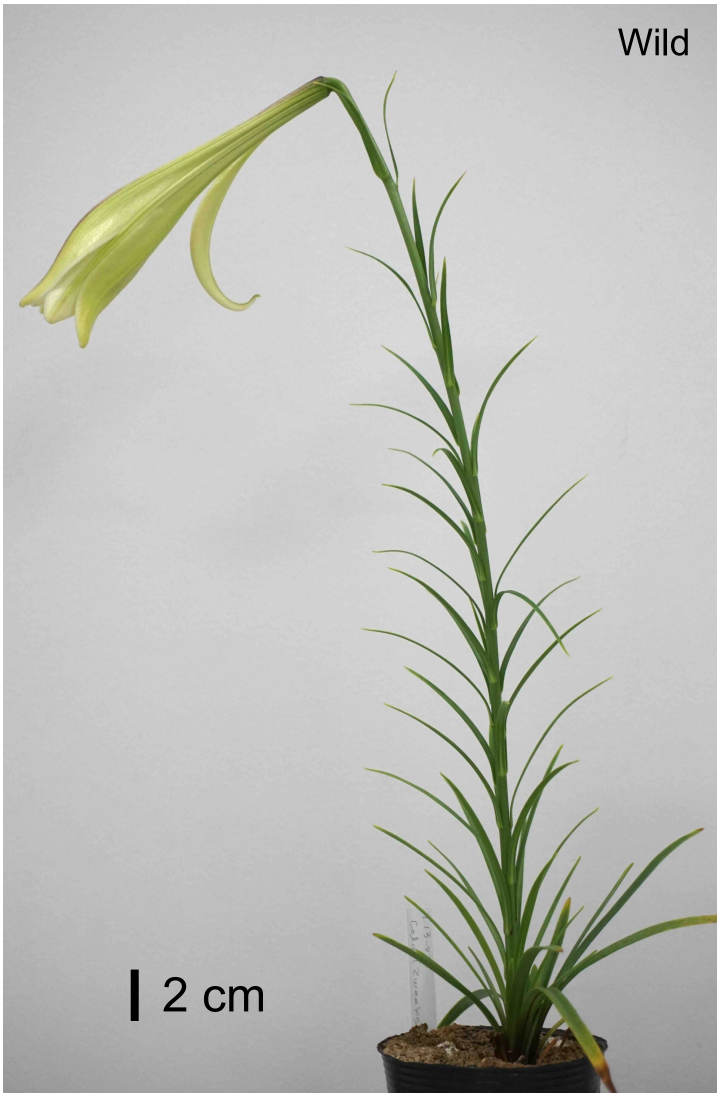

Supplementary Figure S5 Appearances of a callus-derived non-transformed *L. formosanum* plant. Note that this bulb was subject to chilling to stimulate shoot elongation and flowering.
